# Linking ecology, morphology, and metabolism: Niche differentiation in sympatric populations of closely related species of the genus *Littorina* (*Neritrema*)

**DOI:** 10.1002/ece3.7901

**Published:** 2021-07-22

**Authors:** Arina L. Maltseva, Marina A. Varfolomeeva, Roman V. Ayanka, Elizaveta R. Gafarova, Egor A. Repkin, Polina A. Pavlova, Alexei L. Shavarda, Natalia A. Mikhailova, Andrei I. Granovitch

**Affiliations:** ^1^ Department of Invertebrate Zoology St. Petersburg State University St. Petersburg Russia; ^2^ Department of Analytical Phytochemistry Komarov Botanical Institute St. Petersburg Russia; ^3^ Research Park Centre for Molecular and Cell Technologies St. Petersburg State University St. Petersburg Russia; ^4^ Centre of Cell Technologies Institute of Cytology Russian Academy of Sciences St. Petersburg Russia

**Keywords:** cryptic species, ecological niche, ecological speciation, geometric morphometrics, habitat amelioration, *Littorina*, metabolomics, microhabitat distribution, morphological disparity, niche differentiation, shell shape

## Abstract

Divergence of ecological niches in phylogenetically closely related species indicates the importance of ecology in speciation, especially for sympatric species are considered. Such ecological diversification provides an advantage of alleviating interspecies competition and promotes more efficient exploitation of environmental resources, thus being a basis for ecological speciation. We analyzed a group of closely related species from the subgenus *Neritrema* (genus *Littorina*, Caenogastropoda) from the gravel‐bouldery shores. In two distant sites at the Barents and Norwegian Sea, we examined the patterns of snail distribution during low tide (quantitative sampling stratified by intertidal level, presence of macrophytes, macrophyte species, and position on them), shell shape and its variability (geometric morphometrics), and metabolic characteristics (metabolomic profiling). The studied species diversified microbiotopes, which imply an important role of ecological specification in the recent evolution of this group. The only exception to this trend was the species pair *L. arcana* / *L. saxatilis*, which is specifically discussed. The ecological divergence was accompanied by differences in shell shape and metabolomic characteristics. Significant differences were found between *L. obtusata* versus *L. fabalis* and *L. saxatilis* / *L. arcana* versus *L. compressa* both in shell morphology and in metabolomes. *L. saxatilis* demonstrated a clear variability depending on intertidal level which corresponds to a shift in conditions within the occupied microhabitat. Interestingly, the differences between *L. arcana* (inhabiting the upper intertidal level) and *L. compressa* (inhabiting the lower one) were analogous to those between the upper and lower fractions of *L. saxatilis*. No significant level‐dependent changes were found between the upper and lower fractions of *L. obtusata*, most probably due to habitat amelioration by fucoid macroalgae. All these results are discussed in the contexts of the role of ecology in speciation, ecological niche dynamics and conservatism, and evolutionary history of the *Neritrema* species.

## Graphical abstract

Five sympatric *Littorina* (*Neritrema*) species demonstrate clearly different ecological preferences within the intertidal zone (except *L. arcana* and *L. saxatilis* pair). The degree of between‐species niche differentiation correlates with metabolomic characteristics and shell shape differences.

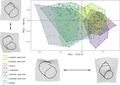

## 1 INTRODUCTION

Ever since the Darwinian description of the Galapagos finches, sympatric populations of phylogenetically close species have attracted the attention of evolutionary biologists. Natural case studies of this kind provide an opportunity to trace the divergence of ancestral species and hypothesize about the drivers of speciation and the mechanisms of emergence of reproductive isolation between nascent species. Notable examples of such model systems are speciation within associations between the host–plant and the phytophagous insect (Futuyma & McCafferty, 1990; Nyman et al., 2006; Peccoud et al., 2010; Percy, 2003, *etc*.), divergence via host switching in parasites (Cox‐Singh, 2012; Duval & Ariey, 2012; Reed et al., 2007, *etc*.), and transition to endosymbiotic lifestyle in microorganisms (Moran & Wernegreen, 2000; Moya et al., 2008). In all these cases, a shift to an alternative niche accompanying species divergence allows the exploitation of different environmental resources even by young species coexisting within the same biocoenosis, that is, sympatric species in a broad sense.

Complexes of sibling species living in sympatry (and, by definition, occupying nonequal niches) are quite common. Their existence highlights the importance of ecology in evolution and the broad occurrence of the so‐called “ecological speciation.” There are two possible scenarios for the formation of such complexes. The first one involves allopatric speciation due to genetic and ecological drift followed by secondary contacts and reinforcement by premating reproductive isolation (Mayr, 1970). The second scenario suggests diversification of ecological niches in situ according to the concept of sympatric speciation (Nosil, 2012; Schluter, 2000). The latter scenario is driven by the ecological specialization of parts of an ancestral population to heterogeneous environments. Genetic processes occurring during sympatric speciation are diverse and cannot be reduced to a single evolutionary pathway (Richards et al., 2019).

Both allo‐ and sympatric ecological speciation models imply niche divergence as a reflection and/or cause of morphological and physiological differences in incipient species. If divergence and speciation events occur, these species expectedly inherit close ecological niches and become ecologically similar. For this reason, ecologically similar species tend to be phylogenetically related (Doenz et al., 2018; Harvey & Rembaut, 2000; McGee et al., 2020; Price, 1997). Numerous observations indicate that some nonrandom mechanisms are responsible for limiting ecological niche expansion during species proliferation (either adaptive or not) and phylogenetic niche conservatism^1^ (Baniaga et al., 2020; Losos, 2008; McGee et al., 2020; Pyron et al., 2015; Wiens & Graham, 2005). Although phylogenetic niche conservatism is a common phenomenon, “niche similarity” is not the same as “niche equivalence” (Warren et al., 2008). The conclusion about the existence of niche conservatism in a particular case depends on the scale of observations (spatial, temporal, environmental, and phylogenetic): Although sister species tend to be more ecologically similar to each other than predicted by random models, their similarity is often not greater than that of more distantly related taxa (Graham et al., 2004; Knouft et al., 2006; Losos et al., 2003; Pearman et al., 2008; Warren et al., 2008). Moreover, stabilizing forces promoting niche conservatism of sympatric species may be opposed by the forces favoring diversification and niche shifts during speciation (Losos, 2008; Losos et al., 2003; Pitteloud et al., 2017; Scriven et al., 2016). From the perspective of population genetics, ecological specialization to different parts of the environment would prevent species from hybridizing and reinforce later divergence (Ackerly, 2003). Such a specialization provides an ecological advantage, alleviating interspecies competition and promoting more efficient exploitation of environmental resources (Gause, 1934; Hardin, 1960; Schluter, 2000).

The present study focuses on the exploitation of different aspects of heterogeneous and dynamic environments within the marine intertidal zone by a set of closely related snail species. Gastropods of the *Littorina* (*Neritrema*) subgenus are a striking example of adaptive radiation within the intertidal zone. On the European North Atlantic shores, the subgenus *Neritrema* comprises two cryptic groups of closely related species—the “obtusata” group (*L. obtusata* [Linnaeus, 1758] and *L. fabalis* [Turton, 1825]) and “saxatilis” group (*L. saxatilis* [Olivi, 1792], *L. arcana* Hannaford‐Ellis, 1978 and *L. compressa* Jeffreys, 1865). Ecotypes of *L. saxatilis* and, to the lesser extent, *L. obtusata* and *L. fabalis* are the best studied *Neritrema* species, while *L. arcana* and *L. compressa* are the least studied because of the difficulties involved in their identification. These species have been examined in many aspects such as morphology and parasitology, phylogeny, genetics and reproductive barriers, adaptive mechanisms, and proteomics (Conde‐Padin et al., 2007; Granovitch et al., 2009; Johannesson, 2003; Maltseva et al., 2016; Maltseva et al., 2020; Panova et al., 2014; Rolán‐Alvarez et al., 2015; Sokolova & Pörtner, 2001a, 2001b, *etc*). Several studies concerning ecological preferences of species or ecotypes of particular species have been conducted (Granovitch et al., 2013; Rolán & Templado, 1987; Sacchi, 1966; Tatarenkov & Johannesson, 1998; Warmoes et al., 1992; Watson & Norton, 1987; Williams, 1995). However, these studies focus on the ecology of one particular *Neritrema* species (or more than one, but never all the five coexisting species) and usually ignore the others, not even mentioning their presence or absence, which might affect the microbiotopic distribution of the species under study. In this way, an important factor is left out of the analysis. While snails of the subgenus *Neritrema* have been used as a model in studies of recent and current speciation (Butlin et al., 2014; Johannesson, 2003, 2016; Johannesson et al., 2017; Morales et al., 2019; Ravinet et al., 2016; Rolán‐Alvarez, 2007; Rolán‐Alvarez et al., 2015), there is still no clear picture of their niche diversification within the complex of sympatric populations.

To fill this gap, we set out to describe the basic parameters of ecological niches of all the five sympatric *Neritrema* species using evidence from their populations in the northeastern Atlantic, where they coexist within the same intertidal zone. We implemented a complex approach with the following characteristics. (a) Two distant sites of the Northern Norway coast (the Barents Sea and the Norwegian Sea) with gravel/stony shores, typical of the northeastern parts of distribution of the studied species, were chosen as models. At these types of coasts, quantitative sampling is possible throughout the intertidal zone. (b) The original sampling method enabled a replicated evaluation of populations’ composition in diverse biotopes regarding tidal level, zonality of macroalgae distribution and open gravel/stony microsites. (c) For the first time, a “layered” sampling was performed to account for the position of the snails in the macroalgal canopy: on the surface, in the depth of the canopy, or on the gravel under it. (d) The species identification was based on the reproductive system morphology to ensure its accuracy. (e) The analysis of the population ecology was supplemented by the geometric morphometric evaluation of shell shape and by metabolomic profiling aimed at revealing the main distinctive physiological features. Such an approach provides an opportunity to reveal unique preferred microhabitats for each species and to match these data with either shell morphology or biochemical characteristics.

*Neritrema* species exhibit a species‐specific microdistribution pattern along the vertical shore gradient (Granovitch et al., 2013), but with a significant overlap: Both *L. arcana* and *L. saxatilis* are present in the fucoid‐free high‐shore zone, while four other species (*L. compressa*, *L. fabalis*, *L. obtusata*, and *L. saxatilis*) inhabit fucoid‐overgrown low shore. We hypothesized that these species should be ecologically segregated within the zones of the overlap. We also tested the possibility of shore‐level‐related shifts in the preferred microhabitat, as different levels provide different sets of microhabitats and coexisting species.

Periwinkles are well known for their adaptive plasticity at both morphological and physiological levels. For example, there are adaptive differences between ecotypes of *L. fabalis* and *L. saxatilis* (reviewed in Johannesson, 2003, 2016; Rolán‐Alvarez, 2007; Rolán‐Alvarez et al., 2015). M and H ecotypes of *L. saxatilis* from British rocky shores are differentiated in relation to wave action: Snails of high‐shore (H) ecotype, which are exposed to a strong wave impact, have a smooth fragile shell with a wide aperture, while snails of *M* (mid‐shore) ecotype have a narrower aperture and a thicker shell wall. Similar tendencies in the shell shape modification depending on the strength of wave action have been shown for exposed and sheltered ecotypes of *L. fabalis*. In various parts of its distribution such as Galicia and Sweden, *L. saxatilis* is known to form a certain ecotype (SU and E, respectively) under strong wave impact, characterized by a small fragile shell with a wide aperture to accommodate a large foot helping to avoid dislodgement (Le Pennec et al., 2017), and a certain ecotype (RB and C, respectively) in the presence of predatory crabs, characterized by a large armored shell with a narrow aperture to withstand crab attacks and excessive desiccation during exposure. Importantly, there is no obvious shore‐level‐related gradient in the strength of wave action on a flat intertidal zone, also, no records of a predatory crab presence at the Northern Norway coasts. Accordingly, we checked the snails under study for the presence of any shell shape changes correlating with the shore level and universal among the species. In particular, we looked for the shell shape changes comparable with those described for ecotypes, such as a globose shell with a wider aperture at the low shore versus a relatively slim elongate shell with a narrow aperture at the high shore, when the action of factors generally accepted as drivers of such morphological changes is absent or very weak.

Morphological specialization contributes much to the adaptation to the microenvironment (e.g., in gastropods, see Vermeij, 1973; Stanley, 1988; Walker & Grahame, 2011; Kistner & Dybdahl, 2013) and seems to be an important part of ecologically based divergence in different organisms (e.g., flies, Filchak et al., 2000; fishes, Doenz et al., 2018; Raffini et al., 2020; palms, Savolainen et al., 2006). Moreover, there are studies implying that the probability of coexistence of morphologically similar species is low (Vodă et al., 2015). Interestingly, no records of distinct ecotypes are available from the northeastern part of the *Neritrema* species distribution ranges, possibly owing to the absence of the predatory crabs at the intertidal zone and the dominance of flat gravel‐stony coastal types. Nevertheless, physiological specialization to contrasting shore levels has been described in *L. saxatilis*. Differences in water conservation abilities, thermal stress resistance, and metabolic rates during low tide have been revealed between *L. saxatilis* snails inhabiting high and low shores (Smith et al., 1995; Sokolova et al., 2000; Sokolova & Pörtner, 2001a, 2001b). Here, we used metabolomic profiling to reveal the species‐specific metabolic background of the snails inhabiting certain microhabitats. Special attention was paid to the compounds of anaerobic metabolism, which are expected to be more abundant in low‐shore snails due to their higher metabolic rate.

We believe that a comparative analysis of the complexes of potentially adaptive traits and ecological preferences, characteristic of closely related snail species, may shed light on their evolutionary history and the role of ecology in their origin. It may also elucidate some fundamental aspects of the mechanisms allowing cryptic species to coexist as well as the dynamics of environmental niches during their evolution.

## 2 MATERIALS AND METHODS

### 2.1 Study sites

Two distant sites at a similar type of the intertidal zone, gravel‐stony with abundant fucoid algae, were chosen for a detailed population analysis of five littorinid species (*L. saxatilis*, *L. arcana*, *L. compressa*, *L. obtusata*, and *L. fabalis)* at coasts of the Norwegian Sea (S1, Saltstraumen, 66°58′10.2″N 13°58′26.5″E, collection dates 29.06–5.07.2019; the average July sea temperature is 13.6℃,^2^ the average length of the day in July is 22 h^2^; the average rainfall in July is 86 mm^2^) and the Barents Sea (V1, Varangerfjord, 70°04′03.9″N 29°58′40.1″E, collection dates 09.07–12.07.2019; the average July sea temperature is 8 °C^2^, the average length of the day in July is 23.8 h^2^; the average rainfall in July is 62 mm^2^). While there are no reports on the *Littorina* species composition in the Norwegian Sea near S1, the set of *Littorina* species in the Barents region has been previously characterized (see, e.g., Galaktionov & Bustnes, 1999; Muraeva et al., 2016; Repkin et al., 2020). Both sites have a flat, moderately exposed gravel‐boulder intertidal zone, about 100 m wide from the lowest to the highest water edge (the map and photographs are in Appendix S1). The middle and the lower part of the intertidal zone at both sites are heavily overgrown by brown macroalgae with a similar species composition. The canopy‐forming macroalgae are key ecosystem engineers within the marine coastal zone, providing habitats for many invertebrates including *Littorina* species. Although the mass of the canopy and the distribution pattern of intertidal fucoids are affected in a complex manner by a number of both abiotic (e.g., wave action) and biotic (e.g., presence of herbivores) factors (e.g., Jonsson et al., 2006; Lappalainen et al., 2019), only the distribution pattern of macroalgae, as the most proximate factor, was used as a proxy to determine the intertidal zonation, which was similar in two sites studied. We followed the generalized zonation scheme of Stephenson and Stephenson (1949, 1972). This scheme does not link the zones to exact tidal heights but determines the relative positions of the major community types across moderately exposed rocky shores (Raffaelli & Hawkins, 1996). The upper part included stones of the littoral fringe and open gravel with sparse clumps of *Fucus vesiculosus*, which do not form a continuous belt or a massive canopy. The lower part was completely covered by macroalgae, with occasional patches of open gravel. The species composition of macroalgae changed seawards in the following manner: *F. vesiculosus* only, *Ascophyllum nodosum* interspersed by *F. vesiculosus*, and *Fucus serratus* interspersed by *F. vesiculosus*.

### 2.2 Microhabitat distribution analysis

The samples were collected at the upper and the lower part of the intertidal zone, the parts of collection being separated by approximately 20 m. At each site, all the samples were collected during the same low tide. There were four types of substrates in the lower part (open gravel, *F. vesiculosus*, *A. nodosum*, and *F. serratus*) and three types of substrates in the upper part (open gravel, stone, and *F. vesiculosus*) (Appendix S1). The samples were collected from haphazardly placed quadrats (0.04 m^2^, 0.20 m × 0.20 m) in five replicates per substrate type (with two exceptions at the lower level on *A. nodosum*, where six samples in Saltstraumen and four in the Varangerfjord were taken). Sampling on fucoids was carried out in layers: the snails were collected separately from the surface of the canopy, from the “depth” of the fucoid canopy, and from the substrate below the canopy. At the upper intertidal level at Saltstraumen, the snails from the depth and the surface of *F. vesiculosus* were collected together and assigned to the depth layer, as there was no clear distinction between these microhabitats. Snails from the open gravel and the fucoid surface were collected immediately from quadrats; then, all the fucoids were cut out and placed into plastic bags together with periwinkles hidden inside for subsequent sorting; finally, the snails were collected from the gravel beneath the canopy (numbers of samples collected for the analysis of microhabitat distribution can be found in Appendix S2).

All the snails were transported to the laboratory for measurements and photodocumentation, followed by dissection for a check for trematode infection, which is known to affect *Littorina* shell shape (Sergievsky & Granovitch, 1999) and for species identification. Snails from the “depth” samples were washed off the algae, which were then weighed. All individuals were dissected under MBI‐10 and Leica EZ4HD stereomicroscopes. Species identification was performed after removing the shell and opening the mantle based on species‐specific characteristics of the reproductive system. Defining features in males included the penis basement and filament form, as well as shape, number, and pattern of penial glands; in females, the decisive characteristics were the morphology of *bursa copulatrix* and the proportions of the auxiliary gland of the pallial complex (for more details, see previous descriptions in: Granovitch et al., 2008, 2013; Maltseva et al., 2016, 2020; Reid, 1996).

### 2.3 Geometric morphometric data

**FIGURE 1:**
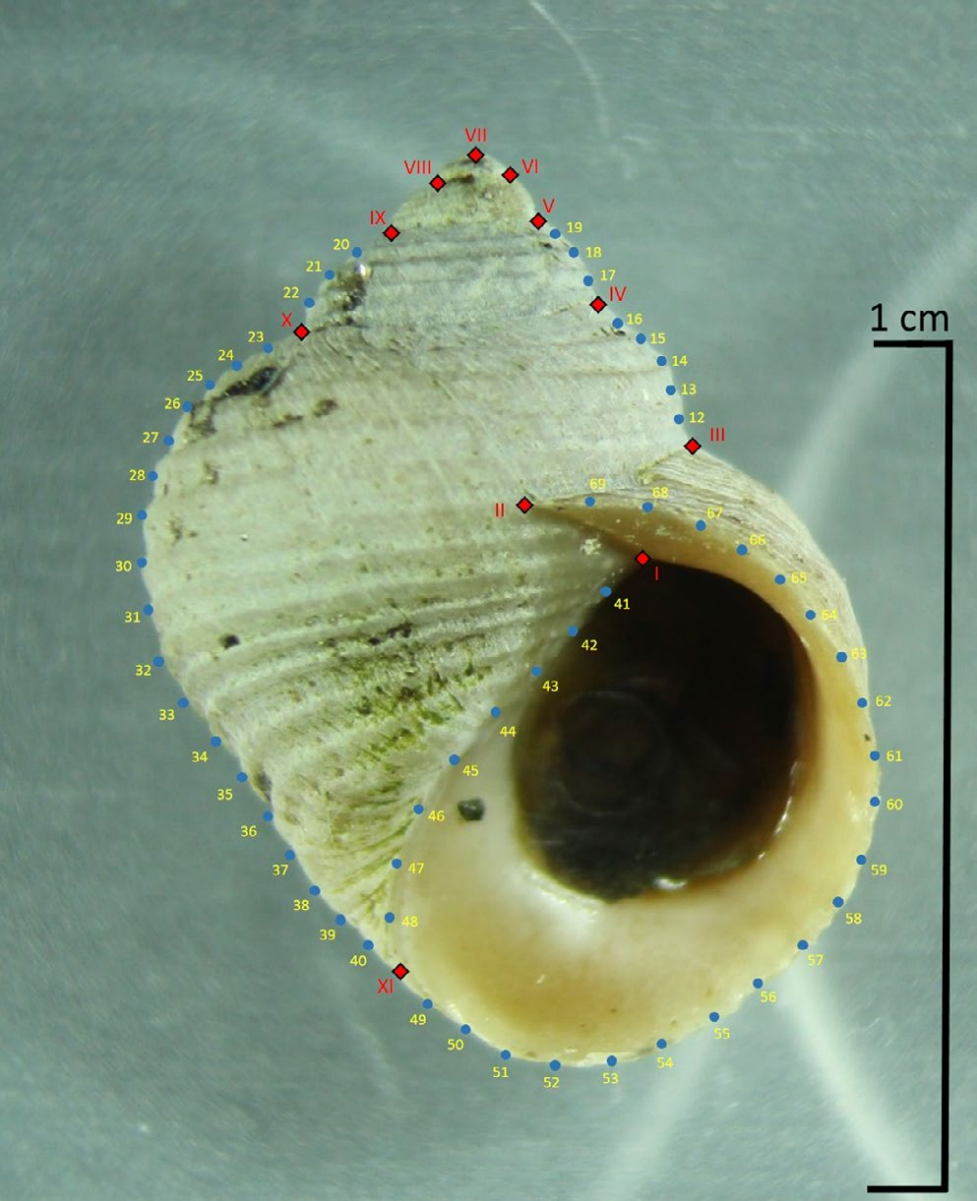
The scheme showing the position of landmarks (I–XI) and semilandmarks (12–69) on periwinkle shells used in this study

Photographic pictures were taken with MBI‐10 binocular microscope coupled with MFU photo adapter and Canon EOS 1200D camera. The camera settings remained unaltered throughout the study. The snails were photographed in a standard position, with the ventral surface of the shell facing up and the aperture in the same plane as the objective, being attached with plasticine for stability. For geometric morphometric analysis, the images were digitized using Tps‐software package (Rohlf, 2015; The Stony Brook Morphometrics); tpsUtil v.1.74 program was used to generate TPS files from two‐dimensional images. The coordinate locations of landmarks and semilandmarks were marked on the images in tpsDig2 v.2.3. The shell shape of the mollusks was described by 11 landmarks and 56 semilandmarks^3^ forming 6 curves (Figure 1). LM1 is in the inner part of the aperture where the parietal wall meets the ultimate whorl; LM2 is at the end of the suture; LM3‐6 are on the right border of the profile of the shell at the end of the suture; LM7 is the apex of the shell; LM8‐10 are on the left border of the profile of the shell at the end of the suture; LM11 is where the outer contour of the columellar lip meets the border of the profile of the shell. In *L. obtusata*, LM6‐8 were located at equal intervals between LM5 and LM9. In total, the analysis was based on 126 individuals of *L. saxatilis* (50–141 mm), 32 individuals of *L. arcana* (81–130 mm), 47 individuals of *L. compressa* (61–113 mm), 128 individuals of *L. obtusata* (55–167 mm), and 41 individuals of *L. fabalis* (50–147 mm); more details can be found in Appendix S3.

### 2.4 Metabolomic data

Metabolomic data were obtained using nontargeted GC‐MS (gas chromatography mass spectrometry)‐based profiling of the trimethylsilyl derivatives. Ninety‐eight adult littorinid snails (28 *L. saxatilis*, 14 *L. arcana*, 14 *L. compressa*, 24 *L. obtusata*, and 13 *L. fabalis*) were collected from the two study sites, an approximately equal number from each site. All the snails were uninfected with parasites and had a well‐developed reproductive system. *L. saxatilis* and *L. obtusata* were collected from both intertidal levels where they occur, 5–7 snails from each level. At each site, the snails were collected during the same low tide, at a similar time moment after air exposure. After collection, the shell and the operculum were removed and the molluskan bodies were individually fixed whole in tubes containing 1 ml of 100% methanol (Merck), frozen at −20℃, and transported to the laboratory, where they were kept at −80℃ until use. Frozen tissues were homogenized in Mixer Mill MM 400 (Retsch) for 20 min at a frequency of 30 Hz with two stainless steel beads (Qiagen). Tissue debris was sedimented by centrifugation at 11 500 g for 15 min; supernatants were collected, vacuum‐dried (CentriVap, Labconco) at 4℃, and frozen at −80℃ until use. Tissue debris was also dried and weighed on Sartorius CPA‐324S analytical balance; weights were used to normalize chromatograms. Before gas chromatography (GC), vacuum‐dried samples (supernatants) were dissolved in 40 µl pyridine (Merck) containing 1 mg/ml of tricosane (nC23) (Sigma‐Aldrich), used as an internal standard for GC‐quantitation, by incubation 10–15 min in ultrasonic bath Elma Elmasonic S100H. Derivatization of analytes by silylation was performed to reduce polarity of compounds (Halket & Zaikin, 2003). For silylation, N,O‐bis(trimethylsilyl)‐trifluoroacetamide with 1% trimethylsilyl chloride (Sigma‐Aldrich) was added to each sample before the analysis. The samples were loaded to glass vials with Teflon septa (Agilent Technologies). The GC‐MS analysis was carried out on a gas chromatograph with a time‐of‐flight mass spectrometer Pegasus 4D GCxGC‐TOF MS (Leco); the carrier gas was helium, and the column was Zorbax DB5 (length 30 m, inner diameter 0.25 mm, film thickness 0.25 µm). The initial temperature was 70℃, the final temperature was 320℃, and the gradient was 6℃ per minute. The injector temperature was 250℃. The scanning frequency was ten spectra per second in a range of masses 50–800 Da. Metabolites were identified using MS‐library NIST10 (National Institute of Standards and Technology) based on match factor and retention indices and using the chromatograms of available standard single amino acids, monosaccharides, fatty acids, and their mixtures. Quantitation of metabolites was performed by the peak total ion current (TIC) on the chromatogram.

### 2.5 Statistical analysis

#### 2.5.1 Analysis of microhabitat distribution

Microhabitat distribution of the periwinkles was analyzed using partial Distance‐based Redundancy Analysis (dbRDA; Legendre & Anderson, 1999; McArdle & Anderson, 2001), based on Bray–Curtis dissimilarities (Bray & Curtis, 1957) on square root‐transformed densities (to reduce the effect of more abundant species on ordination; Legendre & Legendre, 2012), with vegan package (Oksanen et al., 2019) of R (R Core Team, 2020). First, the effect of the microhabitat on the distribution of periwinkles was tested separately in the upper and the lower parts of the intertidal zone; the abundances of the snails sampled from different layers of fucoids were pooled for this analysis. The partial dbRDA model included microhabitat as a predictor (gravel, stone, and *Fucus vesiculosus* in the upper shore; gravel, *F. vesiculosus*, *F. serratus,* and *Ascophyllum nodosum* in the lower shore), and two conditioning variables (site and fucoid weight). Next, the effects of the fucoid species and the position of the snails within the canopy were tested on the macroalgae‐associated samples from the lower part of the intertidal zone. The partial dbRDA model included two predictors: microhabitat (*F. vesiculosus*, *F. serratus*, and *Ascophyllum nodosum*) and layer in the fucoid‐related microhabitat (surface or depth of the fucoid mass and gravel underneath it), and two conditioning variables (site and fucoid weight). To account for nonindependence of layers within a quadrat, it was used as a blocking factor to restrict permutations in tests (which is the way random factors are treated in permutational tests after dbRDA; Oksanen et al., 2019). Finally, the samples taken on *F. vesiculosus*, the only fucoid species present in both the upper and the lower intertidal levels, were used to test the effect of the intertidal level and layer within the fucoid canopy. The model included two predictors: shore level (upper and lower) and layer in the fucoid‐related microhabitat (the same levels as in the previous analysis), and two conditioning variables, site and fucoid weight; permutations were restricted within quadrats. Significance tests were carried out using 9,999 permutations.

#### 2.5.2 Geometric morphometric analysis

Digital images of the shells of the five species of periwinkles were obtained during the survey of microhabitat distribution. Only the photographs of healthy adult snails (no trematode infection, well‐developed reproductive system) were used for geometric morphometric analysis. Statistical analysis was performed in R using geomorph and RRPP packages (Adams & Otárola‐Castillo, 2013; Collyer & Adams, 2018). Landmark coordinates were aligned using the Generalized Procrustes Analysis (Gower, 1975) to remove nonshape variation (effects of position, orientation, and scale). Size (centroid size) and shape (Procrustes coordinates) of each snail were estimated during this procedure. Centroid size was calculated as the square root of the sum of squared distances of landmarks from their centroid.

Measurement error resulting from digitalization of landmarks and semilandmarks was evaluated on a random subsample of 30 snails, which were digitized twice. A Procrustes linear model with an individual as a categorical factor was fitted; mean squares in the resulting ANOVA table were used to calculate the repeatability index as described in Arnqvist and Martensson (1998). A high repeatability index (*R* = 96.5%) indicated a minimal measurements error relative to among‐individual variation.

To visualize shape variation, Procrustes coordinates were subjected to Principal Component Analysis (PCA). Patterns of shape variation were statistically evaluated using nonparametric multivariate analysis of variance (perMANOVA; Anderson, 2001) with randomized residual permutation procedure (RRPP; Collyer & Adams, 2018) on the matrix of Procrustes coordinates. The model for interspecific comparisons of the shape of all five littorinid species included the following predictors: species/subpopulation (a categorical predictor with seven levels defined by species and subpopulation: the upper or the lower shore for *L. saxatilis* and *L. obtusata*), a random effect of collection site, *log*‐transformed centroid size (to account for possible allometric effects; Bookstein, 1991), and all two‐ and three‐way interactions of these factors. In addition, separate models with the same set of predictors were fitted for shape comparisons within “saxatilis” and “obtusata” species groups. Predicted shell shapes for species/subpopulations were obtained from these models at the mean centroid size of the respective group to correct for allometric changes. Shell shape predictions were visualized using thin‐plate spline transformation grids. Morphological disparity was quantified for different species and their subpopulations from the upper and the lower intertidal levels. It was estimated as the Procrustes variance using residuals of the model (with the function pairwise() in the package geomorph). 95% bias‐corrected and accelerated (BCa) confidence intervals for morphological disparities were estimated using a nonparametric bootstrap (DiCiccio & Efron, 1996) with 999 iterations using the package boot (Canty & Ripley, 2020; Davison & Hinkley, 1997). Post hoc comparisons of mean shapes, allometric vectors, and morphological disparities among species/subpopulations were done using the above‐mentioned models for “saxatilis” and “obtusata” species groups. The analyses were performed after accounting for the site‐specific allometric differences, *that is*, the null model, which was used to extract residuals for RRPP included *log*‐centroid size, collection site, and their interaction. The tests were performed using 999 permutations. *p*‐values in post hoc tests were corrected for multiple testing using Holm–Bonferroni procedure (Holm, 1979). Sample sizes in these analyses were unbalanced. Less accurately estimated means (Cardini & Elton, 2007) and larger variation in small samples (Tversky & Kahneman, 1971) may lead to an overestimation of the differences in small groups from the larger ones (Cardini et al., 2015). Thus, the differences between *L. arcana*, whose sample size was the smallest (altogether 32 snails: 8 in Saltstraumen and 24 in Varangerfjord), and the other species should be treated with caution.

#### 2.5.3 Metabolomic analysis

Raw metabolomic data (detection intensities) were log‐transformed, normalized by sample weight (NormalizeMets package; De Livera & Olshansky, 2018), and quantile normalized (limma package; Ritchie et al., 2015). The metabolomes were visualized using nonmetric multidimensional scaling (nMDS) based on Euclidean distances among the samples (vegan package; Oksanen et al., 2019). Interspecies differences among metabolomes were tested using perMANOVA with 9,999 permutations in vegan package, after testing for the assumption of homogeneity of within‐group dispersions, which was met. To note, the sample sizes were unbalanced in this analysis, but perMANOVA is robust to sample imbalance in the absence of heterogeneity of within‐group dispersions (Anderson & Walsh, 2013). The sets of metabolites that best distinguished the species were obtained using principal least squares discriminant analysis (PLS‐DA; Barker & Rayens, 2003) using mixOmics package (Rohart et al., 2017). The concentration of several anaerobic metabolites such as succinate, lactate, and malate was compared in *L. saxatilis* and *L. obtusata* snails from different intertidal levels using the moderated *t* test (Smyth, 2004) in the limma package (Ritchie et al., 2015). The same comparison was performed for *L. arcana* and *L. compressa*. The moderated *t* test is more powerful than an ordinary *t* test, especially for small samples, because it moderates the sample variances for each metabolite using the information on the distribution of sample variances across all metabolites with the help of an empirical Bayes method.

## 3 RESULTS

### 3.1 Ecological mapping

*Littorina* (*Neritrema*) species composition differed in the upper and lower intertidal zones. *L. fabalis* was registered only in the lower part, while *L. arcana* was registered only in the upper part. The upper and lower parts of the intertidal zone also differed as to the dominant species of fucoid macroalgae and their projective cover. Only *F. vesiculosus* was present in the upper part, with a patchy distribution and a relatively low projective cover (0%–25% in any quadrat within the upper shore area). The lower part was almost completely covered by three species of fucoids (*F. vesiculosus*, *F. serratus*, and *A. nodosum*), and patches of open sediment were rare and scattered.

#### 3.1.1 The upper intertidal zone

**FIGURE 2:**
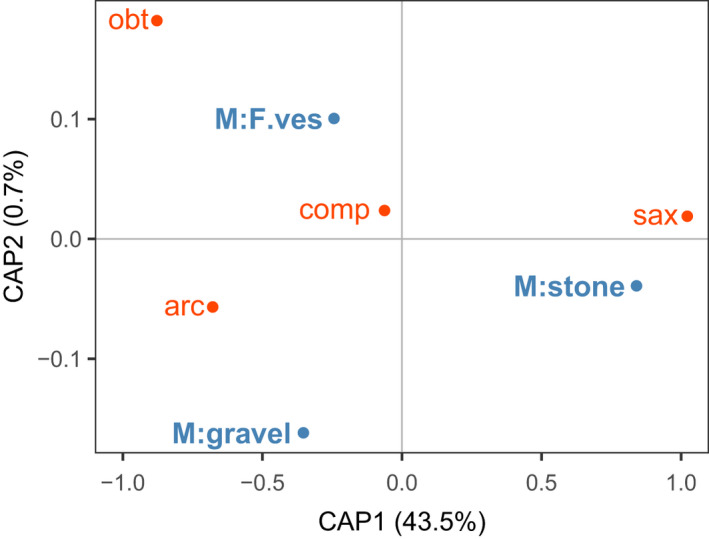
Distribution of the *Littorina* species across microhabitats in the upper littoral zone. The effect of microhabitat on littorine species composition was analyzed after controlling for between‐site differences using partial distance‐based redundancy analysis (dbRDA). Bray–Curtis dissimilarities were calculated from the square roots of species abundances in samples. Microhabitats: open stones (M:stone), open gravel (M:gravel), clumps of *Fucus vesiculosus* (M:F.ves); *Littorina* species: arc—*L. arcana*, comp—*L. compressa*, obt—*L. obtusata*, sax—*L. saxatilis*. The relative position of the microhabitat centroids (blue) and species (red) reflects the predominant spatial distribution of the snails in the intertidal zone

Four species of periwinkles (*L. arcana*, *L. compressa*, *L. obtusata,* and *L. saxatilis*) inhabited three microhabitats (open stones, open gravel, and *F. vesiculosus* clumps) in the upper intertidal. The effect of microhabitat on species composition was significant after controlling for between‐site differences (permutational test after partial dbRDA, *p* < .001; Appendix S2) and canonical axes constrained by this factor together explained 44.2% of the total variation. *L. arcana* and *L. saxatilis* mainly occupied open microniches (gravel and stones), *L. arcana* tended to be more common on gravel, and *L. saxatilis*, on stones (Figure 2). *L. compressa* and *L. obtusata* showed an “attraction” to *F. vesiculosus*.

#### 3.1.2 The lower intertidal zone

**FIGURE 3:**
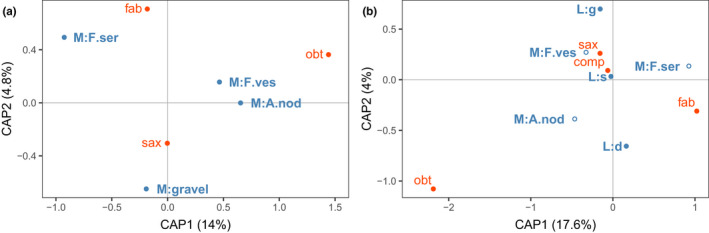
Distribution of the *Littorina* species across microhabitats in the lower intertidal zone. The partial dbRDA ordinations explore differences in *Littorina* species composition (a) across microhabitats and (b) stratification in the fucoid‐related microhabitats after controlling for between‐site differences. The analyses were based on Bray–Curtis dissimilarities calculated from the square roots of species abundances in samples. Microhabitats: open gravel (M:gravel), *F. serratus* (M:F.ser), *A. nodosum* (M:A.nod), *F. vesiculosus* (M:F.ves); layer in the fucoid‐related microhabitat: surface of the fucoid mass (L:s), depth of the fucoid mass (L:d), gravel under the fucoid mass (L:g), *Littorina* species: comp—*L. compressa*, fab—*L. fabalis*, obt—*L. obtusata*, sax—*L. saxatilis*. The relative position of the microhabitat centroids (blue) and species (red) reflects the predominant spatial distribution of the snails in the intertidal zone

Harbored a different set of the four *Littorina* species (*L. compressa*, *L. fabalis*, *L. obtusata,* and *L. saxatilis*) in four principal microhabitats (*F. vesiculosus*, *F. serratus*, *A. nodosum*, and open gravel) than the upper intertidal zone. We performed a two‐step partial dbRDA analysis of the littorine distribution after controlling for between‐site variation: across all microhabitats when snails from different layers of the fucoid canopy were pooled (Figure 3a) and across a subset of fucoid‐related microhabitats accounting for their layered structure (surface, depth of the fucoid canopy, and gravel under it; Figure 3b). The canonical axes in the resulting models explained 20.1% and 22.3% of total variation, respectively.

Microhabitat type significantly affected the composition of *Littorina* species at the lower intertidal level (*p* < .001 in both analyses; Appendix S2). Two species, *L. obtusata* and *L. fabalis*, were strongly associated with fucoid macroalgae: the former with *A. nodosum* (slightly stronger) and *F. vesiculosus*, and the latter with *F. serratus*. Conversely, *L. saxatilis* was most commonly registered in open gravel microhabitats or, to a lesser extent, associated with *F. vesiculosus* (Figure 3a). The layer of fucoid‐associated habitats also significantly affected *Littorina* species distribution (*p* ≤ .05, Appendix S2). *L. obtusata* was usually buried in the depth of the fucoid mass, while *L. fabalis* was equally abundant in the depth and on the surface (Figure 3b). *L. saxatilis*, when associated with *F. vesiculosus*, kept on the gravel under the fucoid canopy. The fourth species, *L. compressa*, was predominantly associated with the surface of *F. vesiculosus* and was also found, albeit very rarely, in open gravel microhabitats.

#### 3.1.3 Association with *F. vesiculosus* in different levels

**FIGURE 4:**
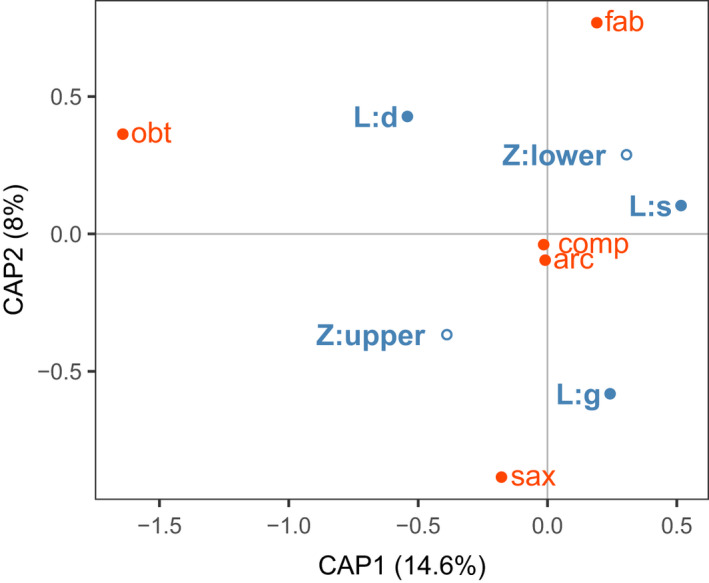
Distribution of the *Littorina* species in association with *Fucus vesiculosus*. The dbRDA ordination explores variation in the distribution of littorines within *F. vesiculosus* clumps in the upper and lower parts of the intertidal zone after accounting for between‐site differences. The analysis was based on the Bray–Curtis dissimilarities on square roots of species abundances in samples. Shore level: upper (Z:upper) or lower (Z:lower) and stratification by layers of the fucoid‐related microhabitats: surface of fucoid mass (L:s), depth of the fucoid mass (L:d), gravel under fucoid mass (L:g); *Littorina* species: arc—*L. arcana*, comp—*L. compressa*, fab—*L. fabalis*, obt—*L. obtusata*, sax—*L. saxatilis*. The relative position of the microhabitat centroids (blue) and species (red) reflects the predominant spatial distribution of the snails in the intertidal zone

*Fucus vesiculosus* is the only fucoid macroalga present both in the lower and upper parts of the intertidal zone. All the five *Littorina* species were associated with it to some degree. In the *F. vesiculosus* samples, the variation of littorine species composition across shore levels was not significant after controlling for between‐site variation; in contrast, the effect of the layer within *F. vesiculosus* canopy was significant (partial dbRDA, *p* = .23 and *p* < .001, respectively; Figure 4, Appendix S2). Taken together, these factors explained 25.5% of the total variation.

*L. arcana*, when associated with *F. vesiculosus* at the upper level, was present only on the gravel below the canopy, while *L. compressa* occurred at both shore levels, either inside algal clumps or on the gravel beneath them. *L. fabalis* was present in the lower part only and was associated with both the surface and the depth of *F. vesiculosus* canopy. *L. obtusata* and *L. saxatilis* displayed association with this fucoid macroalgae species predominantly in the upper part of the littoral zone. The former species was detected usually within the depth of the fucoid canopy and the latter on the gravel beneath it.

### 3.2 Shell morphology

**FIGURE 5:**
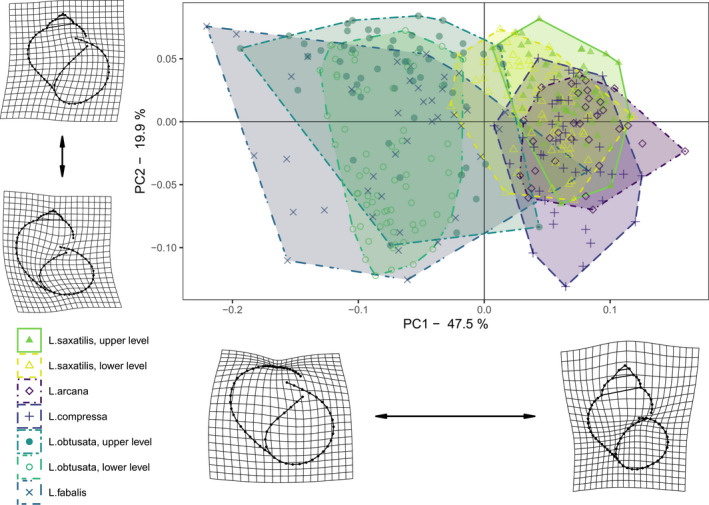
Shell shape variation in five *Littorina* species. Principal component analysis ordination of shell shapes grouped by species and subpopulation (upper or lower intertidal level). Thin‐plate spline transformation grids represent shell shape changes between the mean shape and minimum and maximum values of PC1 and PC2. For clarity, the magnitude of shell shape changes on the positive end of PC1 was reduced 0.6 times

It is often regarded as an adaptive trait in snails (Kistner & Dybdahl, 2013; Stanley, 1988; Vermeij, 1973; Walker & Grahame, 2011). As *Littorina* species studied do differ as to the occupied microhabitats, we evaluated possible interspecific shell shape differences and the level of intraspecies variability. When we visualized the shell shape variation using PCA, the first principal component explained almost a half of the total variability, indicating prominent differences between the “saxatilis” and “obtusata” species groups in the relative shell width, general globosity, and spire height (Figure 5). The Procrustes linear model confirmed the significance of “species/subpopulation” (upper or lower intertidal levels), “collection site”, and interaction of these factors. There was no effect of sex on shell shape, except Saltstraumen population of *L. compressa* (Appendix S3). Due to the significant allometric effect and its interactions, the correction for the site‐specific allometric differences was included in all the subsequent analyses (more details are in Appendix S3).

#### 3.2.1 In the “saxatilis” species group

Shell shape depended strongly on site (Procrustes linear model, *p* = .001, standardized effect size Z = 6.4; Appendix S3) and its interaction with species/subpopulation (*p* = .001, Z = 5.6), which indicates site‐specific differences among species/subpopulations. Allometric relationships did not significantly differ between species/subpopulations (*p* = .241, Z = 0.7) and were site‐specific (*p* = .001, Z = 2.6). The species‐specific allometries did not differ between sites (*p* = .119, Z = 1.1).

**FIGURE 6:**
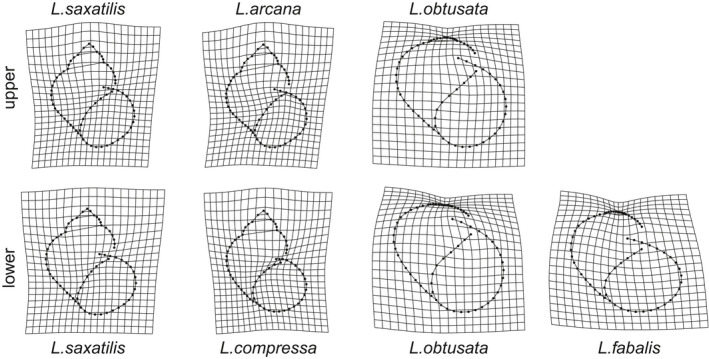
Predicted mean shapes of five *Littorina* species. Predictions for “saxatilis” and “obtusata” groups were made from separate Procrustes linear models, both of which included species/subpopulation, site, log‐centroid size, and their interactions. Shell shapes averaged across sites were predicted at the mean centroid size of the corresponding species group to correct for allometric changes. Thin‐plate spline transformation grids reflect modification of shell shape relative to the overall mean shape. For clarity, shell shape changes were magnified 1.5 times

Average shell shapes of *L. compressa* significantly differed from those of *L. arcana* and *L. saxatilis* from both shore levels after site‐specific allometric differences had been removed (Figure 6, Table 1). Its predicted shell shape was slimmer and had a more rounded aperture than that of the other species of the “saxatilis” group (Figure 6). The strongest shape differences in average shapes were found between *L. compressa* and *L. saxatilis* from the upper and the lower intertidal levels (*p* = .001 in both cases, Z = 9.8 and Z = 6.3, correspondingly; Table 1). The differences between *L. arcana* and *L. saxatilis* were weak and not significant (Figure 6, Table 1), those between *L. arcana* and upper subpopulations of *L. saxatilis* being the weakest (*p* = .175, Z = 0.9). Comparison of allometric angles yielded a similar pattern of differences (Table 2; Appendix S3). Based on the model predictions, *L. saxatilis* at the lower level, similarly to *L. obtusata*, tends to possess a more widely open aperture and a generally more globose shell (Figure 6, Table 1).

**TABLE 1:** Post hoc comparisons of mean shell shape between the *Littorina* (*Neritrema*) species

Contrast	d	UCL (95%)	Z	*p*	*p* adj.
*L. arcana : L. compressa*	**0.047**	**0.032**	**4.6**	.**001**	.**006**
*L. arcana : L. saxatilis_high*	0.032	0.036	0.9	.175	.175
*L. arcana : L. saxatilis_low*	0.042	0.042	1.8	.052	.123
*L. compressa : L. saxatilis_high*	**0.053**	**0.021**	**9.8**	.**001**	.**006**
*L. compressa : L. saxatilis_low*	**0.046**	**0.025**	**6.3**	.**001**	.**006**
*L. saxatilis_high : L. saxatilis_low*	0.024	0.022	2.0	.041	.123
*L. fabalis–L. obtusata_high*	**0.067**	**0.037**	**6.4**	.**001**	.**003**
*L. fabalis–L. obtusata_low*	**0.083**	**0.048**	**6.6**	.**001**	.**003**
*L. obtusata_high–L. obtusata_low*	**0.041**	**0.031**	**3.4**	.**005**	.**005**

Note

Analyses within the “saxatilis” and “obtusata” groups were performed independently. Regional allometric differences had been removed. Significantly differing contrasts are written in bold.

Abbreviations: d, pairwise distances between means; *p adj*., adjusted *p*‐values; *p*, *p‐*values; UCL (95%), upper confidence limit; Z, standardized effect size.

**TABLE 2:** Post hoc comparisons of the direction of allometric shape change in the *Littorina* (*Neritrema*) species

Contrast	*r*	Angle	UCL (95%)	Z	*p*	*p* adj.
*L. arcana : L. compressa*	**−.332**	**109.40**	**105.22**	**1.9**	.**035**	.**175**
*L. arcana : L. saxatilis_high*	.736	42.59	68.95	−0.04	.463	1.0
*L. arcana : L. saxatilis_low*	−.061	93.49	127.35	−0.5	.679	1.0
*L. compressa : L. saxatilis_high*	**−.382**	**112.45**	**87.96**	**3.4**	.**002**	.**012**
*L. compressa : L. saxatilis_low*	.117	83.30	84.04	1.8	.055	.220
*L. saxatilis_high : L. saxatilis_low*	.424	64.90	89.02	−0.7	.749	1.0
*L. fabalis–L. obtusata_high*	.825	34.43	42.74	0.6	.261	.783
*L. fabalis–L. obtusata_low*	.909	24.60	39.88	−0.7	.737	.930
*L. obtusata_high–L. obtusata_low*	.947	18.81	28.62	<0.1	.465	.930

Note

Analyses within the “saxatilis” and “obtusata” groups were performed independently. Regional allometric differences had been removed. Significantly differing contrasts are written in bold.

Abbreviations: angle, angles between vectors; deg, the correlation between the vectors is the cosine of the angle between the vectors (the smaller the angle, the larger the correlation); *p adj*., adjusted *p*‐values; *p*, *p*‐values; *r*, pairwise correlations between vectors of shape change; UCL (95%), upper confidence limit; Z, standardized effect size.

The angles between allometric relationships of *L. compressa* also differed significantly from those of *L. arcana* and *L. saxatilis* from the upper shore level after site‐specific allometric differences had been removed (Table 2). The difference between *L. compressa* and *L. saxatilis* from the upper intertidal level was the strongest (*p* = .002, Z = 3.4; Table 2), while the difference between *L. compressa* and the lower shore *L. saxatilis* was weaker and not significant (*p* = .055, Z = 1.8; Table 2). *L. compressa* had the highest morphological disparity among the species of the “saxatilis” group, which significantly differed from *L. arcana* and *L. saxatilis* from both shore levels (Appendix S3).

#### 3.2.2 In the “obtusata” species group

The shell shape was significantly affected by the collection site (Procrustes linear model, *p* = .001, Z = 7.1; Appendix S3) and by the interaction with species/subpopulation factor (*p* = .001, Z = 4.9), which was similar to the situation observed in the “saxatilis” group of species. Site‐specific allometric shape changes were significant (*p* = .001, Z = 4.9); while species allometries did not differ significantly (*p* = .303, Z = 0.5), the species‐specific allometric relationships significantly differed between sites (*p* = .002, Z = 2.9).

Average shell shapes of *L. fabalis* significantly differed from *L. obtusata* of both shore levels (*p* = .001 in both cases) after site‐specific allometric differences had been removed (Table 1, Figure 6). These shape differences were much stronger than the differences between *L. obtusata* from the upper and the lower intertidal levels (Z = 6.4 and Z = 6.6, respectively, versus Z = 3.4, *p* = .005). The shell of *L. fabalis* has a more expanded outer lip and a more rounded aperture (Figure 6). Angles between allometric relationships of *L. fabalis* did not significantly differ from those of *L. obtusata* from both shore levels after site‐specific allometric differences had been removed (Table 2; Appendix S3). *L. fabalis* had a significantly higher morphological disparity than *L. obtusata* from the upper and lower shore levels (*p* = .001 in the both cases, Z = 5.3 and Z = 6.4, correspondingly; Appendix S3). Importantly, there were no significant differences between the upper and lower fractions of *L. obtusata* populations (Z = −0.5, *p* = .638; Tables 1 and 2).

*Littorina obtusata* individuals with strongly deformed shells, with a pressed spire and a right‐angle fin on the body whorl or, vice versa, with a very high spire, were registered in the Varangerfjord population. Their frequency was approximately the same at the upper and lower levels: 7.2% and 6.3%, respectively. Both these forms of monstrosity were described by Reid (1996). No such forms were found in the Saltstraumen population of *L. obtusata* and in both populations of *L. fabalis*.

### 3.3 Physiological variation among species and subpopulations

**FIGURE 7:**
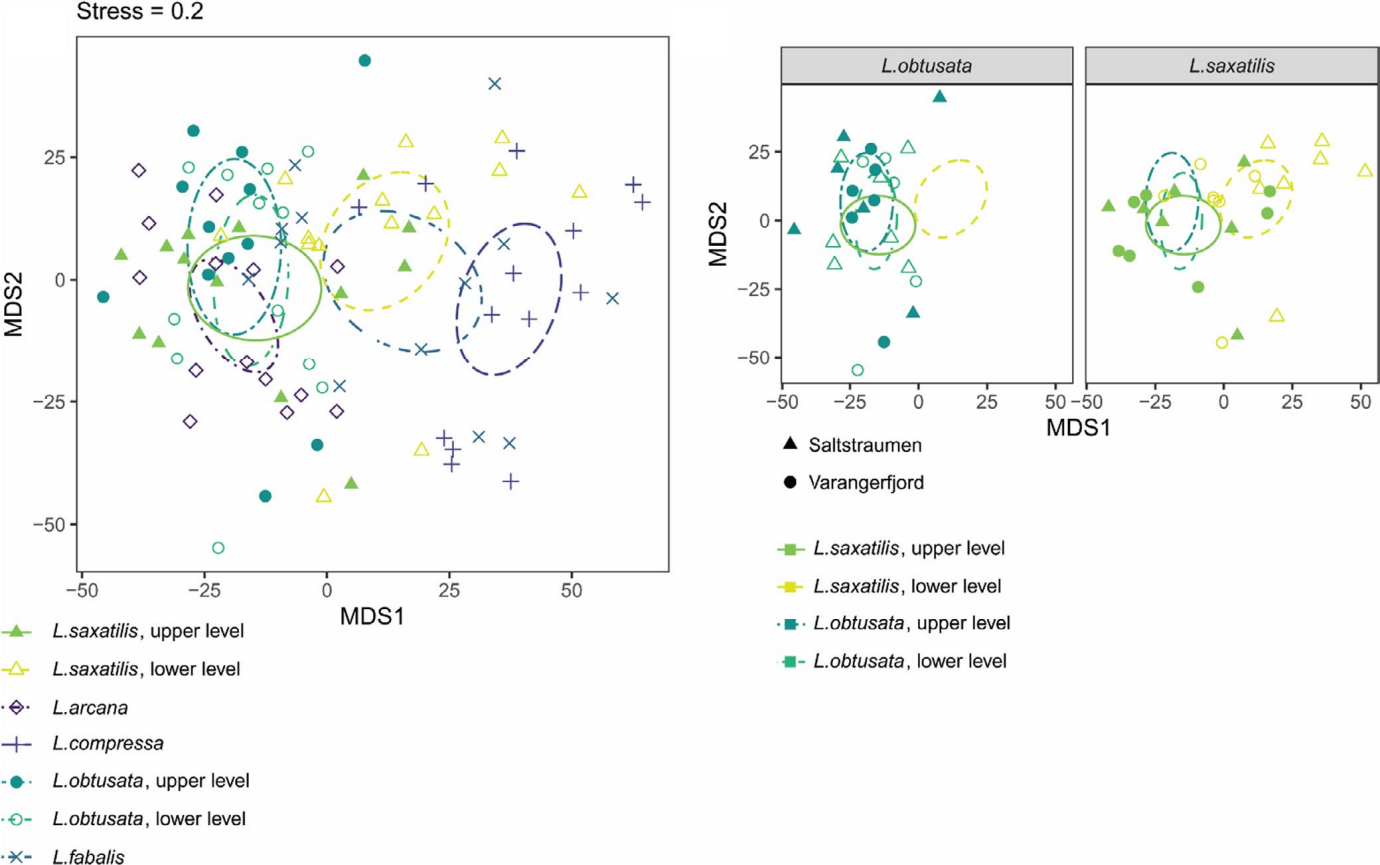
nMDS ordination of whole metabolomes based on dry weight and quantile normalized quantitative data and selected component comparison. (a) ordination of whole metabolomes by species, 95% confidence intervals of centroids are visualized. (b) ordination of whole *L. saxatilis* and *L. obtusata* metabolomes regarding collection site and intertidal level, 95% confidence intervals of centroids are visualized

We applied a metabolomic approach to juxtapose physiology and niche differentiation of the *Littorina* species. We analyzed “instantaneous snapshots of metabolism” as all the samples from each site were collected in parallel during the same low tide. The metabolomes varied significantly across species/subpopulations (*p* = .0001, perMANOVA) and sampling sites (*p* = .0001), the pattern of variation differed at different sites (interaction was significant, *p* = .0002) (Figure 7, Appendix S5).

#### 3.3.1 Interspecific variation

Significant differences were detected between the species of the “obtusata” group, *L. fabalis* and *L. obtusata*, at both intertidal levels (Figure 7a). Interspecies differences were also registered within the “saxatilis” group (Figure 7a). There was no overlap in the distribution of metabolomes between *L. arcana* and *L. compressa* (*p* = .002, post hoc perMANOVA), while the metabolome of *L. saxatilis* overlapped both with that of *L. arcana* and, to a much lesser extent, with that of *L. compressa*. The fraction of *L. saxatilis* overlapping with *L. arcana* was mainly represented by high‐shore inhabitants (see Appendix S5 and below). As a result, these two species were metabolically indistinguishable at the upper intertidal level. At the same time, the differences between *L. compressa* and *L. saxatilis* from both levels as well as between *L. arcana* and lower shore *L. saxatilis* were significant (*p* = .002, post hoc perMANOVA). Species‐distinguishing metabolites, according to PLS‐DA, were mainly represented by saturated and unsaturated fatty acids, cholesterol, adenosine, tryptophan, and pantothenic acid (the full list is given in Appendices S4 and S6).

#### 3.3.2 Shore‐level effects

**FIGURE 8:**
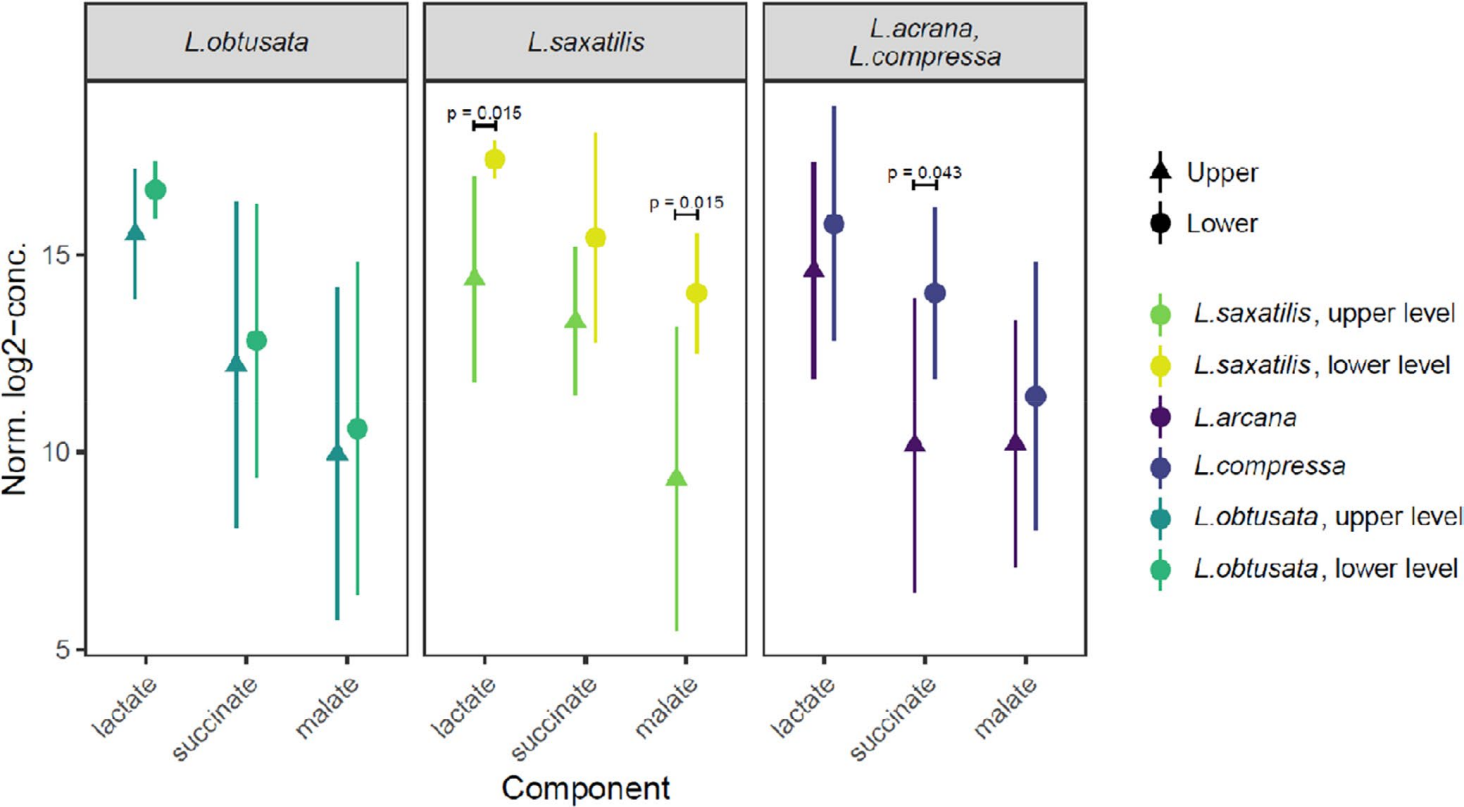
Comparison of abundances of anaerobic metabolites (in detection intensity units) at different collection sites and intertidal levels. Means and 95% confidence intervals are shown. *p*‐values were obtained from the moderated *t* test (Smyth, 2004)

Two species, *L. saxatilis* and *L. obtusata*, from, respectively, the “saxatilis” and the “obtusata” cryptic group were collected from two intertidal levels. The shore level did not significantly affect the metabolome of *L. obtusata* but did affect that of *L. saxatilis* (Figure 8b). The differences in *L. saxatilis* metabolomes at different shore levels were more prominent in Saltstraumen than in Varangerfjord.

Since air exposure during low tide is known to onset the anaerobic metabolic pathways, the concentrations of anaerobic metabolic compounds were specifically analyzed. These compounds did not differ significantly in *L. obtusata* from different intertidal levels (Figure 8, Appendix S6). At the same time, in *L. saxatilis*, lactate and malate were significantly more abundant in lower shore snails (*p* = .015, moderated *t* test; Figure 8), with the increase in the succinate, though insignificant, showing the same tendency. Finally, succinate was significantly more abundant in *L. compressa* than in *L. arcana* (*p* = .043, moderated *t* test; Figure 8), with malate and lactate showing the same tendency. Amino acids involved in anaerobic metabolism, aspartate and alanine, demonstrated no clear tendency in any of the species studied (Appendix S6).

## 4 DISCUSSION

In this study, we evaluated the shell morphology and the metabolomes in a group of closely related sympatric *Littorina* (*Neritrema*) species in the context of occupied microhabitats. All the five *Neritrema* species studied at the intertidal zone of the Barents and the Norwegian Sea coasts clearly demonstrated niche conservatism (the whole family Littorinidae includes more than 200 exclusively inter‐ and supratidal species; Bouchet et al., 2005). Nevertheless, some niche specialization and differentiation between species are expected, as phylogenetically related species usually occupy similar but not equivalent niches (Losos, 2008; Pitteloud et al., 2017; Scriven et al., 2016; Wiens & Graham, 2005). The prevalence of niche divergence over niche conservatism is stronger for sympatric species due to such mechanisms as interspecies competition alleviation and efficient exploitation of environmental resources (Losos et al., 2003; Pitteloud et al., 2017; Schluter, 2000; Silvertown et al., 2001). Here, we present a detailed study of microhabitat distribution of five coexisting littorinid species, demonstrating their niche nonequivalence to provide possible adaptive interpretations of observed differences in shell morphology and physiology. Based on our results, we cannot conclude whether the occupied microhabitats belong to their optimal range. We can just pinpoint the fact of their ecological segregation, owing to either differential adaptive scope, or typical biotic associations, or competition, or a combination of these factors. Nevertheless, inhabiting a certain type of microhabitat requires morphological and physiological adaptations, which are a precondition of ecology‐driven speciation. Thus, along with microdistribution patterns, we analyzed morphological and physiological differences between the *Neritrema* species and inferred their possible adaptive relevance.

### 4.1 Ecology

*Littorina obtusata* seems to be the most ecologically distinct species. It preferentially occupies the depth of the fucoid canopy: *A. nodosum* in the lower part of the littoral zone and *F. vesicolosus* in the upper part. *L. fabalis* is associated with another fucoid, *F. serratus*, occupying mainly surface of the fucoid canopy. This difference in localization between the two “obtusata” species concords with the results of an earlier study, which showed that *L. obtusata* was negatively phototactic and tended to retreat into the algal mass while *L. fabalis* actively crawled on the algal surface during air exposure (Guiterman, 1970).

Two species from the “saxatilis” group, *L. saxatilis* and *L. arcana*, inhabit stony and gravel substrates, mainly in the upper part of the intertidal zone. The former spreads lower down the littoral on the gravel under *F. vesiculosus*. *L. compressa* seems to be an “ecological stranger” within the “saxatilis” group. It preferentially dwells in the lower part of the intertidal zone in association with *F. vesiculosus*, on the surface and beneath the fucoid mass. Unexpectedly, individuals of *L. arcana* were occasionally registered in a similar microhabitat. These results demonstrate a clear partitioning of the environments by five closely related periwinkle species.

Species of the “obtusata” group favor different “host” alga species, and it was probably this “host” switch that promoted the divergence of *L. fabalis* and *L. obtusata*. Moreover, *L. obtusata* is not only epiphytic but also phytophagous, feeding on the thalli of *Fucus* and *Ascophyllum* and displaying resistance to their toxic polyphenols, while *L. fabalis* prefers to graze on bacterial films on the surface of the thalli (Norton et al., 1990; Norton & Manley, 1990; Reid, 1996). In the “saxatilis” group, *L. saxatilis,* besides grazing solid substrates, is known to feed on seedlings of the green alga *Enteromorpha* but avoids feeding on brown algae (Lotze & Worm, 2000), which corresponds to our observation of its preferred occurrence under the fucoid canopy in the lower level. Thus, although the distribution of *L. obtusata* partially overlaps with that of both *L. fabalis* and *L. saxatilis* during low tide, these three species are ecologically separated: *L. obtusata* and *L. fabalis* inhabit clumps of various macroalgae, while *L. saxatilis* occupies gravel under the macroalgal canopy.

*Littorina compressa* is the only species of the “saxatilis” group tightly associated with fucoid macroalgae. Being a micrograzer, *L. compressa* keeps to *F. vesiculosus*, probably scraping the bacterial film from its surface, as *L. fabalis* does on the surface of *F. serratus*. Similarly to *L. saxatilis*, *L. compressa* spatially overlaps with *L. obtusata* but their niches are different due to the specificity of feeding. On the whole, when the microenvironment is taken into account, there is no obvious phylogenetic signal (correspondence between phylogenetic relatedness and ecological niche similarity; Blomberg & Garland, 2002) in ecological preferences of the studied species, as *L. compressa*, being a species of the “saxatilis” group, demonstrates a pattern more typical of “obtusata” species. This observation is in line with the rapid rate of ecological divergence between sympatric species and the absence of a clear correlation between the ecological similarity of sympatric species and their phylogenetic proximity (Losos et al., 2003; Warren et al., 2008).

It should be emphasized that the habitat preferences of the periwinkles described in this paper are not universal. For example, *L. obtusata* appears to prefer *F. vesiculosus* to *A. nodosum* as “host” species and feeding substrate in Scotland (Watson & Norton, 1987) but favors *A. nodosum* in populations of Wales and Norway (Williams, 1995). *L. fabalis* ecotypes in the Iberian Peninsula are associated with either *Fucus vesiculosus*, or *Zostera* spp. or *Mastocarpus (as Gigartina) stellatus*, depending on the wave exposure of a coast (Reid, 1996; Rolán & Templado, 1987). In Sweden, *L. fabalis* is found throughout the eulittoral zone on the open stones or in the canopy of *F. serratus*, *F. vesiculosus*, *F. spiralis*, and *A. nodosum*, not being confined to the lower littoral part (Tatarenkov & Johannesson, 1998). At the same time, in Wales and Scotland, the distribution pattern of *L. fabalis* is very similar to the one described here (Reimchen, 1981; Watson & Norton, 1987). Even when only moderately exposed coasts are compared, ecological preferences of *L. fabalis* (and other species, as expected) vary in Galicia, Sweden, and Norway. To be more precise, the ecological preferences of each species form a unique pattern in every particular coast type. Nevertheless, the analysis of a variety of the distribution patterns could be used for understanding these preferences.

Another important point to keep in mind is a temporal variation of the environmental regime in the littoral zone: Intertidal organisms are regularly emerged and submerged during the tidal cycle. Besides typical emerged and submerged states in all littoral inhabitants, there is a specific regime of their change and specific mechanisms regulating the physiological transition between these two states. Ecological niche characteristics include parameters of emerged and submerged conditions and transitions between them, requiring adequate adaptations. Furthermore, there are fluctuations in temperature, humidity, salinity, *etc*., even if only submerged or emerged state analyzed due to day–night and seasonal variations. Irregular variability also occurs. In our study, only one dimension of this complex system was analyzed—“the instantaneous ecology and physiology of emerged state.” This description, though incomplete, is undoubtedly informative as it allows one to outline at least a subset of possible stressors and adaptations to withstand them.

### 4.2 Shell morphology

We found that shell morphology was highly variable in three of the studied species: *L. obtusata*, *L. fabalis*, and *L. saxatilis*. In the latter two species, shell shape polymorphism has been found to be associated with shore conditions such as wave exposure, temperature stress, and crab predation, and contrasting ecotypes have been identified (Reid, 1996). In *L. obtusata*, a significant variability in shell shape has been noted (Reid, 1996) but no ecotypes have been recognized. To note, two geographical morphs, the “northern” turbinate *palliata* and the “southern” globose *retusa*, have been described in *L. obtusata* (Reid, 1996). *L. fabalis*, though similar in shell shape to *L. obtusata* and forming similar geographical morphs, the “northern” turbinate *limata* and the “southern” globose *typica*, is more patulous and has a thicker columellar lip (Reid, 1996). A hypothetical explanation is that these differences reflect a stronger ability of *L. fabalis* to attach to a substrate and thus to occupy more exposed habitats (Tatarenkov & Johannesson, 1998; Williams, 1990, 1992, 1995). In line with this hypothesis, a geometric morphometric analysis showed significant shell shape differences between *L. obtusata* and all three *L. fabalis* ecotypes in the Iberian Peninsula (Carvalho, 2014). Our morphometric analysis confirmed that in Norway the shell of *L. fabalis* differs from that of *L. obtusata*, having a more expanded aperture. This probably means that *L. fabalis* has a more massive foot, which is consistent with the above hypothesis.

Contrasting ecotypes of *L. saxatilis* in environmental gradients of rocky shores have been extensively studied (e.g., Carvajal‐Rodriguez et al., 2005; Faria et al., 2019; Galindo et al., 2010; Johannesson et al., 1997; Ravinet et al., 2016; Rolán‐Alvarez, 2007, *etc*.). They are usually represented by either a globose shell with a wide aperture, which is better suited for withstanding wave impact, or a higher, often thickened and armored shell with a narrower aperture, which allows the snail to endure higher temperature/desiccation stress and/or to resist the attacks of crabs (Johannesson, 2003; Mill & Grahame, 1995; Rolán‐Alvarez et al., 2015). In this study, we found similar tendencies in the predicted mean shapes of *L. saxatilis* and *L. obtusata*, though they were weak for the former and weaker still for the latter species (Figure 7). Our study sites were not steep rocky but flat gravel‐stony, with a moderate wave action and no expected gradient in this action along shore height; additionally, there are no predatory crabs in the area. This is probably the reason behind the weaker differences between the high‐shore and the low‐shore subpopulations found in our study. Nevertheless, the factor underlying the presence of these differences is still of high interest. In an experimental work of Kemp and Bertness (1984) on *L. littorea*, similar shell shape changes were not adaptive but were caused by differences in metabolic and growth rates. Snails living on poor diet developed elongate shell with narrow aperture, while the mollusks on a rich diet had thin globose shell with expanded aperture (Kemp & Bertness, 1984). It seems self‐consistent that periwinkles of high‐shore have less ample feeding regime due to a longer exposure during the low tide. Thus, shore level‐related difference in shell morphology observed in the studied *Neritrema* species may be partially nonadaptive but neutral and caused by different allometric patterns.

In our study, *L. arcana* most closely resembled the higher fraction of *L. saxatilis* both in shell shape and microhabitat preferences, as well as in metabolome characteristics and the proteome (Maltseva et al., 2016, 2020). This contradicts earlier studies of this species pair based on linear morphometric analysis, which has shown not only significant differences between sympatric *L. arcana* and *L. saxatilis* in British populations but also the nonequivalence of *L. saxatilis* depending on the presence of *L. arcana* (Grahame & Mill, 1989). *L. arcana* has a more open aperture than *L. saxatilis* and has been reported to possess a larger foot (Grahame & Mill, 1986). It has been hypothesized that these features explain the ability of *L. arcana* to occupy more exposed sites and substrates than *L. saxatilis* in Britain (Dytham et al., 1990). The discrepancy between our results and earlier studies might be due to the differences between the coast types: strongly exposed in Britain versus moderately exposed in Norway. However, it is more likely that it results from specific features of the British *Littorina* populations, which have a long history of isolation from the mainland ones (Doellman et al., 2011; Maltseva et al., 2020; Panova et al., 2011). Indeed, other morphometric studies of British periwinkles revealed shell shape differences in *L. saxatilis* and *L. arcana*, comparable with the differences between either of them and *L. compressa* (Caley et al., 1995; Conde‐Padin et al., 2007).

According to our results, *L. compressa* significantly differed from *L. arcana* and *L. saxatilis* in the shell shape, while the latter two species were barely distinguishable in this respect, especially if only the upper fraction of *L. saxatilis* was taken into account. The shell of *L. compressa* is relatively high and slim, with a small round aperture (Figure 7) but it is difficult to find any adaptive background in this. These differences correspond to our long‐term field experience, which suggests that *L. compressa* may be recognized based on shell morphology, and not only the shape but also the texture of the shell (our observations, unpublished), and to the results of the metabolomic analysis.

### 4.3 Metabolome similarity

Instantaneous metabolic “snapshots” were obtained to describe the physiological status of the periwinkles under study and to trace a correlation with their ecological preferences. In general, the metabolomes of these phylogenetically close littorinid species were, expectedly, rather similar (Figure 7). Nonetheless, there were significant interspecies differences, while the effect of the intertidal level was less certain. We have found no metabolomic (and no shell shape) differences between the upper and lower fractions of *L. obtusata* populations, but found robust differences between *L. obtusata* and *L. fabalis*. The latter two species occupy ecologically different microhabitats (in terms of intertidal level, preferred species of fucoid macroalgae, and position in/on “host” fucoid during low tide), which may explain the observed differences in metabolomes (here) and proteomes (Maltseva et al., 2020). *L. obtusata* switches a preferred “host” macroalgae from *A. nodosum* on the lower/mid‐shore to *F. vesiculosus* on the upper shore, where the former fucoid is absent. Robert McMahon suggested the existence of two adaptive strategies in intertidal gastropods (McMahon, 1990). Inhabitants of the eulittoral zone (the low‐ to mid‐shore zone) struggle to retain metabolic activities even when air‐exposed during low tide, while the dwellers of the littoral fringe (the high‐shore zone) slow down metabolic processes and isolate themselves by closing shells with opercula. We suggest that the upper and lower subpopulations of *L. obtusata* do not differ significantly in metabolomic features due to habitat amelioration by fucoid macroalgae: The microclimate within a canopy of the “host” fucoid remains relatively stable during the tidal cycle, even though the fucoids are different at different levels. Thus, *L. obtusata* physiologically represents eulittoral fauna even in the upper part of the shore in association with *F. vesiculosus*.

Unlike *L. obtusata*, *L. saxatilis* showed significant variation of the metabolome depending on the intertidal level. This result is consistent with many previous studies on the vertical subdivision of *L. saxatilis* populations (not considering contrasting ecotypes) in neutral genetic markers (Sokolov et al., 2003), general proteomic features (Maltseva et al., 2016), water conservation abilities, behavior, and metabolic enzymes stability and productiveness (Smith et al., 1995; Sokolova et al., 2000; Sokolova & Pörtner, 2001a, 2001b). In particular, regarding the last point, it was shown that the allele of a less productive aspartate aminotransferase (AAT) enzyme is more common among the upper shore *L. saxatilis* snails (Panova & Johannesson, 2004). AAT catalyzes transamination reaction during the transition to anaerobic metabolism, which leads to the accumulation of end products of diverse pathways of pyruvate transformation (with tissue‐dependent intensity): succinate, malate, and lactate (De Zwaan, 1983; Livingstone & De Zwaan, 1983; Sokolova et al., 2000). Metabolic depression in the upper shore *L. saxatilis* fraction during low tide was confirmed in the White and North Sea populations (Sokolova et al., 2000; Sokolova & Pörtner, 2001a, 2001b). Accordingly, in our study, the average concentrations of succinate, malate, and lactate were slightly higher in the metabolomes of the lower level *L. saxatilis* snails (Figure 8, for more details, see Appendix S6). Notably, there was no such clear tendency in the case of *L. obtusata* (Figure 8). This confirms the existence of two different adaptive strategies in *L. saxatilis* snails from the upper and the lower shore levels (littoral fringe and eulittoral following McMahon, 1990) in contrast to *L. obtusata*.

The differences between the other two species of the “saxatilis” group, *L. arcana* (the species of the littoral fringe) and *L. compressa* (the species of the eulittoral zone), tended to be similar to those between the upper and the lower fractions of *L. saxatilis* (Figure 8). This concerns both a set of metabolites differentiating the upper and the lower shore *L. saxatilis* and anaerobic metabolism compounds (Appendix S6). Such metabolome properties are well consistent with the ecological preferences of these species. Both *L. arcana* and *L. saxatilis* inhabit stony and gravel biotopes on the upper shore, which are prone to acute desiccation, temperature variation, and other stressors. At the same time, both *L. compressa* and *L. saxatilis* are associated with *F. vesiculosus* in the low shore, where environmental conditions are milder. Interestingly, the metabolome of *L. compressa* still differed from that of the lower *L. saxatilis* (Appendix S6), probably reflecting the difference in their microhabitats: gravel under the fucoid canopy (*L. saxatilis*) versus the surface of the fucoid mass (*L. compressa*).

Our results, supplemented by the published data, provide a backbone to the reconstruction of the niche divergence and the evolution of species‐specific features in the complex of phylogenetically close snail species. The estimated divergence time between “saxatilis” and “obtusata” groups is ~3.25–2.83 Mya (Reid et al., 1996, 2012). The event was probably related to the specialization of flat periwinkles (“obtusata” group) to association with and feeding on brown macroalga (fucoids) against rough periwinkle *L. islandica*, a presumable ancestor of the three species of the “saxatilis” group, grazing bacterial biofilms from solid substrates (Reid, 1996). The feeding specialization was accompanied by a modification of the radula, which is clearly different in grazers on macroalgae (“obtusata”) compared with micrograzers of the “saxatilis” group (Reid, 1996). Differences in shell morphology between the species groups also became apparent: more globose shells in “obtusata” versus coniform shells with pronounced concaved suture in “saxatilis.”

Diversification within groups seems to be due to inhabiting different microhabitats, which can be partially characterized through their typical position during low tide. *L. obtusata* and *L. fabalis* developed an association with different fucoid macroalgae: *A. nodosum* and *F. vesiculosus* versus *F. serratus*, respectively. The “host” algae, in turn, determined their distribution along the vertical intertidal gradient (*L. obtusata* occupies a higher zone than *L. fabalis*) as well as the complexes of morphological and physiological adaptations. The differences in the “host” alga species, the position on it, the preferred feeding substrate, and the distribution pattern (differential regimes of air exposure, wave action, *etc*.) are reflected in clear differences in the metabolomes and the shell morphology.

Among the “saxatilis” species, *L. saxatilis* represents a typical generalist, being able to spread from the upper to the lower intertidal levels, inhabit both sheltered and exposed shores, and enter estuarine areas. Owing to this, it has the widest geographical range in the subgenus (Reid, 1996). This species is associated with gravel, open at the upper intertidal level and covered by fucoid macroalgae at the lower one. This microhabitat shift is linked to different regimes of exposure to air and extreme temperatures, desiccation, and other stressors. These differences underlie shore level‐dependent variation in shell shape and metabolomic characteristics, illustrating the ability of this species to form partially isolated specialized subpopulations. In contrast, *L. obtusata* populations occupy a fairly stable microhabitat within the fucoid canopy, which weakens level‐dependent effects. *L. compressa* is tightly associated with *F. vesiculosus*, and this association seems to limit the distribution of this species to the uppermost part of the intertidal zone and to determine its metabolomic and conchological peculiarity. *L. arcana* closely resembles *L. saxatilis* at the upper intertidal level in shell shape, metabolome, and ecological preferences. Some fraction of *L. arcana* descends into the lower part of the intertidal zone in association with *F. vesiculosus*. This fraction was not analyzed in this study, but is expected to differ from the upper part, similarly to the upper and the lower shore *L. saxatilis* based on our previous results (Maltseva et al., 2016). Moreover, some laboratory and field observations suggest the possibility of a restricted gene flow between *L. arcana* and *L. saxatilis,* the existence of presumable hybrids, and interspecies copulations in wild populations (Granovitch et al., 2013; Maltseva et al., 2021; Mikhailova et al., 2009; Warwick et al., 1990). Thus, the divergence between these two species is of particular interest since the ecological specification can hardly be its driving force. Some postmating prezygotic reproductive isolation mechanisms, *for example*, those associated with polymorphism of gamete interaction proteins, could be expected to function in this pair of species and to contribute to their nonecological and nongeographical speciation (Lobov et al., 2018; Maltseva et al., 2021).

## 5 CONCLUSIONS

The *Neritrema* species are a vivid example of niche nonequivalence between sympatric close relatives, although in general they retain niche conservatism, remaining within the intertidal zone. We detected a clear differentiation of potentially adaptive traits related to shell morphology and metabolite composition between species with clearly delineated microhabitats. On the other hand, there was no such differentiation of the metabolomes and the shell morphology between the species inhabiting the same biotope in the high‐shore (*L. saxatilis* and *L. arcana*). These findings accentuate the role of adaptation in the divergence of the Northern Atlantic *Neritrema* species and provide a rationale for considering them as a living example of recent ecological speciation, with the exception of *L. arcana*/*L. saxatilis* pair. The driving force of divergence in the latter species pair can become a hot spot of future research.

## CONFLICT OF INTEREST

None declared.

## AUTHOR CONTRIBUTIONS

**Arina L. Maltseva:** Conceptualization (lead); Data curation (lead); Methodology (lead); Supervision (equal); Validation (equal); Writing‐original draft (lead). **Marina A. Varfolomeeva:** Data curation (equal); Methodology (lead); Software (lead); Validation (equal); Visualization (lead); Writing‐original draft (equal). **Roman V. Ayanka:** Data curation (supporting); Methodology (equal); Software (supporting); Visualization (supporting); Writing‐original draft (supporting). **Elizaveta R. Gafarova:** Methodology (equal); Writing‐original draft (equal). **Egor A. Repkin:** Methodology (equal); Writing‐original draft (equal). **Polina A. Pavlova:** Methodology (equal); Writing‐original draft (equal). **Alexei L. Shavarda:** Methodology (lead); Writing‐original draft (supporting). **Natalia A. Mikhailova:** Conceptualization (equal); Methodology (equal); Supervision (supporting); Writing‐review & editing (supporting). **Andrei I. Granovitch:** Conceptualization (lead); Data curation (equal); Funding acquisition (lead); Methodology (equal); Project administration (lead); Supervision (equal); Validation (equal); Writing‐review & editing (lead).

### OPEN RESEARCH BADGES

This article has earned an Open Data Badge for making publicly available the digitally‐shareable data necessary to reproduce the reported results. The data is available at [https://doi.pangaea.de/10.1594/PANGAEA.923735].

## Supporting information

Appendix S1

Appendix S2

Appendix S3

Appendix S4

Appendix S5

Appendix S6

## Data Availability

Microhabitat distribution (counts in quantitative samples), shell shape (TPS files with landmark positions and metadata), and metabolome compositions (normalized detection intensities) will be available at Pangaea.de (https://doi.org/10.1594/PANGAEA.923735).

## ENDNOTES

^1^
Phylogenetic niche conservatism is a phenomenon of closely related species being more similar ecologically than would be expected by simple Brownian motion (Wiens & Graham, 2005; Losos, 2008).

^2^
Climatic data from the Weather Atlas https://www.weather‐atlas.com.

^3^
Landmarks are the anatomical loci that can be reliably defined on the structures studied (Bookstein, 1991). The same set of landmarks should be recognised on all specimens. Semilandmarks are used to describe the shape of curves where it is impossible to identify discrete anatomical loci repeatedly. Semilandmarks are series of points located at equal intervals along the curves (Bookstein, 1997).
