# Supplementary material for: Linking ecology, morphology, and metabolism: Niche differentiation in sympatric populations of closely related species of the genus Littorina (Neritrema)
Source: Ecol Evol. 2021 Jul 22;11(16):11134–54. doi: 10.1002/ece3.7901 (PMC8366845; doi:10.1002/ece3.7901)

Linking ecology, morphology and metabolism: niche differentiation in sympatric populations of closely related species of the genus *Littorina* (Neritrema)

Arina L. Maltseva[1], Marina A. Varfolomeeva[1], Roman V. Ayanka[1], Elizaveta R. Gafarova[1], Egor A. Repkin[1], Polina A. Pavlova[1], Alexei L. Shavarda[2,3], Natalia A. Mikhailova[1,4], Andrei I. Granovitch[1]

1 Department of Invertebrate Zoology, St. Petersburg State University, St. Petersburg, Russia
2 Department of Analytical Phytochemistry, Komarov Botanical Institute, St. Petersburg, Russia
3 Research Park, Centre for Molecular and Cell Technologies, St. Petersburg State University, St.-Petersburg, Russia
4 Centre of Cell Technologies, Institute of Cytology Russian Academy of Sciences, St. Petersburg, Russia

Appendix_1. Details on collection sites.

A1 Fig_1. The map of the sites studied (image: TerraMetrics, map data: Google). Sample collection sites are shown: S1 Saltstraumen, Norway, 66°58'10.2"N 13°58'26.5"E, collection dates 29.06-5.07.2019; V1 Varangerfjord, Norway, 70°04'03.9"N 29°58'40.1"E, collection dates 09.07-12.07.2019.

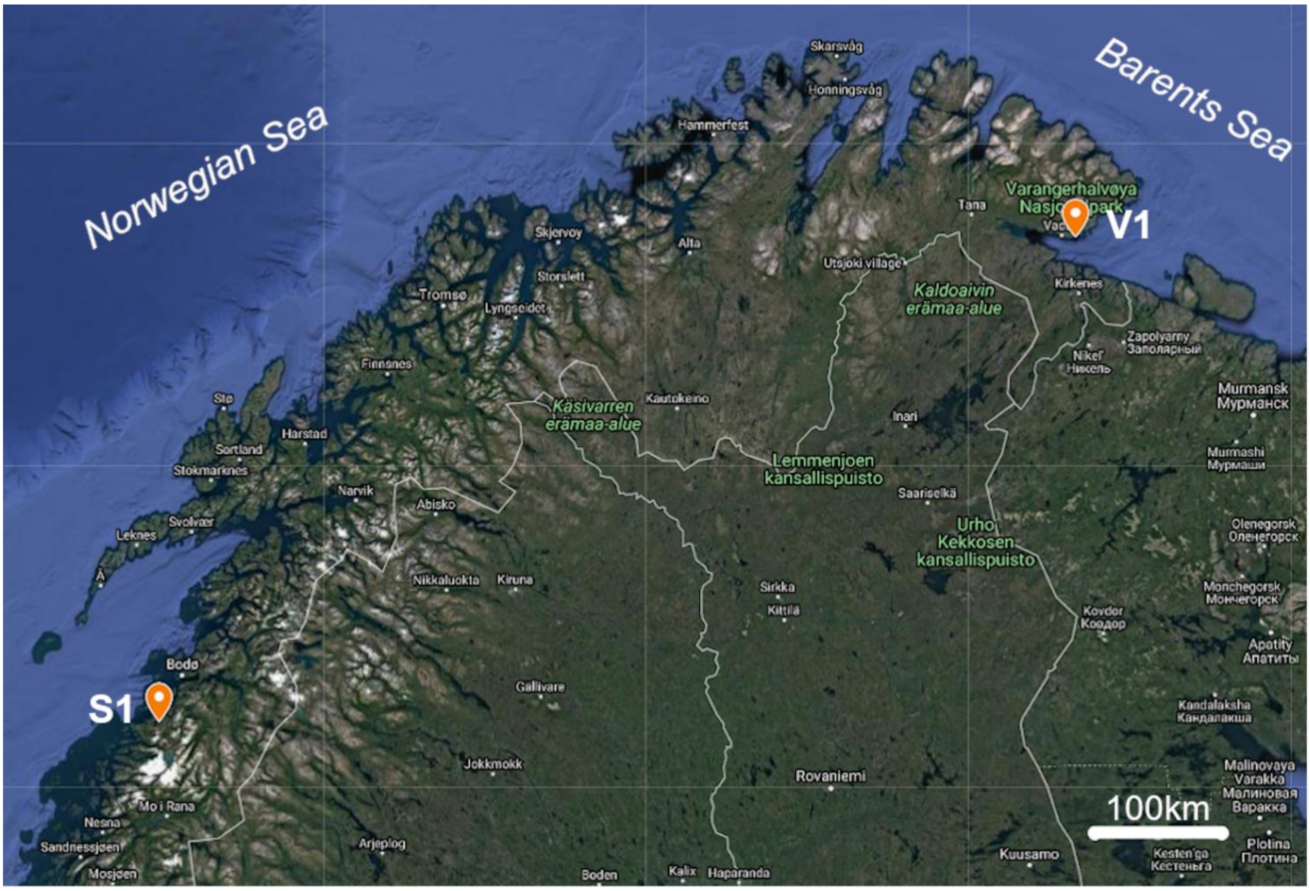

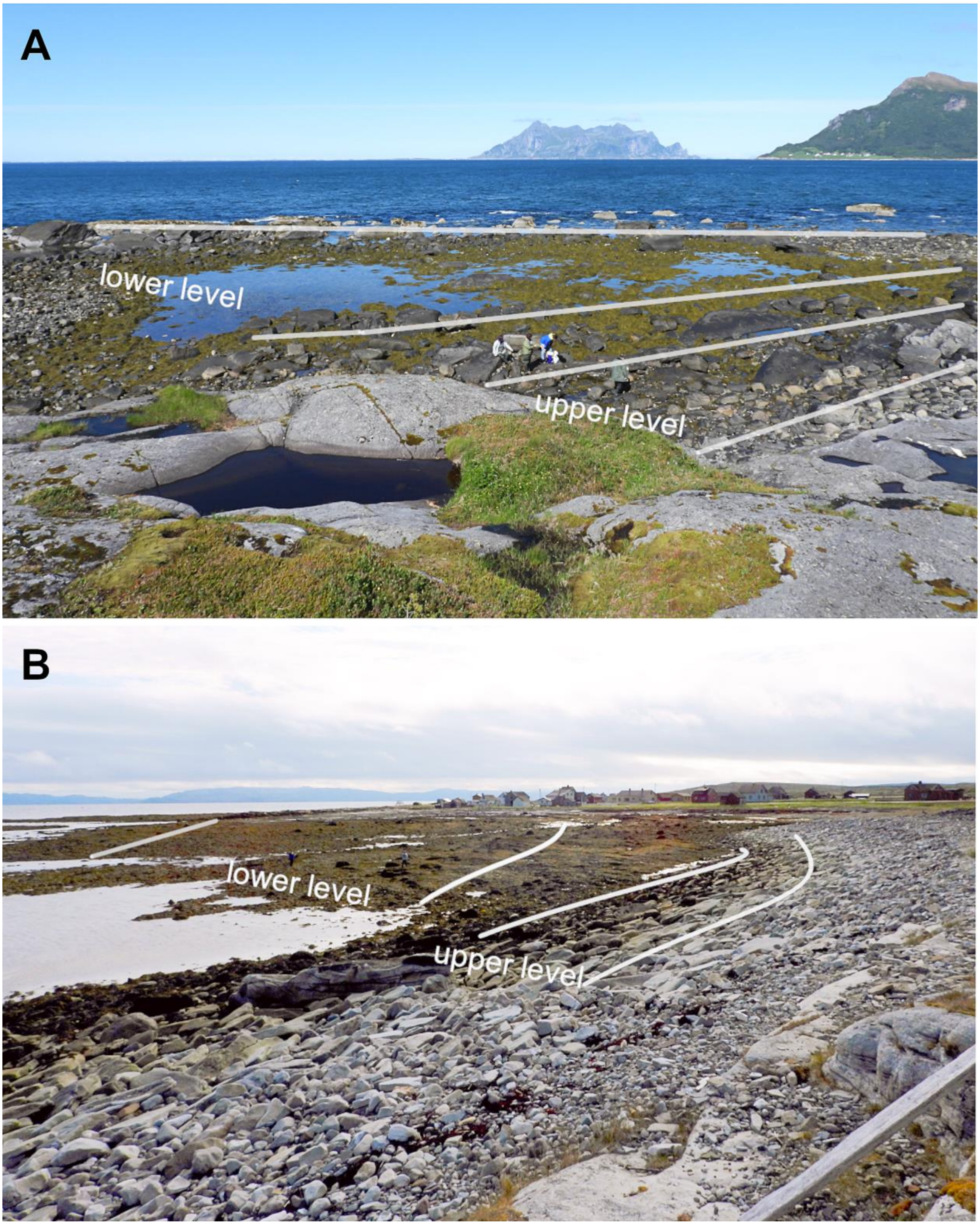

A1 Fig_2. The littoral zone of the collection sites. Intertidal levels were determined by macroalgae distribution. The upper level included stones free of macrophytes and sparse clumps of *Fucus vesiculosus*. The lower level was extensively overgrown by macroalgae (*F. vesiculosus*, *Ascophyllum nodosum*, *F. serratus*), forming a continuous belt. There was no contact zone between the upper and lower levels. No obvious gradient in the strength of wave action is expected at the flat stony-gravel intertidal zone due to very small slope angle and, consequently, a significant width (up to 100-150 m) . S1: Saltstraumen site; V1: Varangerfjord site. The coast of S1 is somewhat more exposed to wave action than V1-site, since the latter is located at the side of the fjord (partially closed water area).

A1 Fig_3. Sampling scheme. Types of microhabitats sampled in the upper and lower intertidal levels and the layered sampling scheme in the clumps of fucoid algae.

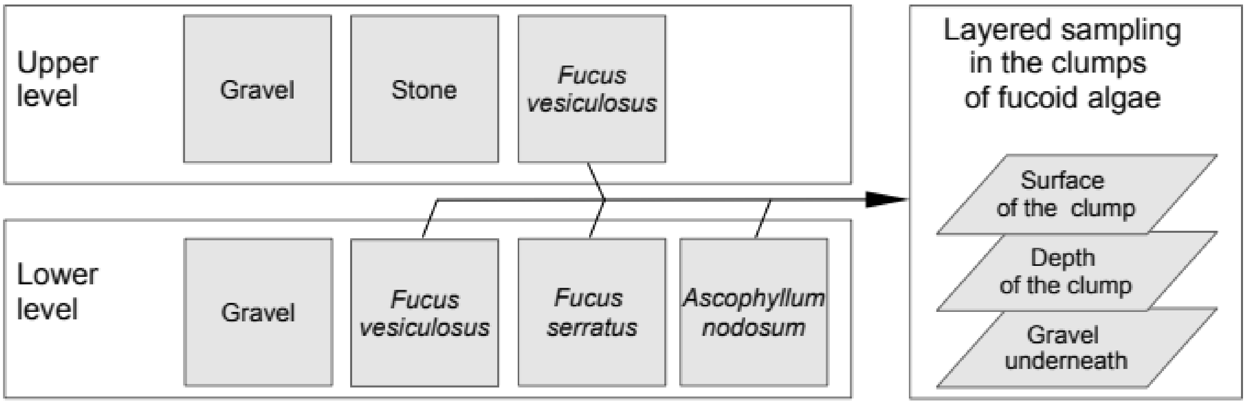

Supplement: Supplementary file 1 — Appendix S1 [file ECE3-11-11134-s002.pdf]
