# Supplementary material for: Linking ecology, morphology, and metabolism: Niche differentiation in sympatric populations of closely related species of the genus Littorina (Neritrema)
Source: Ecol Evol. 2021 Jul 22;11(16):11134–54. doi: 10.1002/ece3.7901 (PMC8366845; doi:10.1002/ece3.7901)
Supplement: Supplementary file 2 — Appendix S2 [file ECE3-11-11134-s003.pdf]

Linking ecology, morphology and metabolism: niche differentiation in sympatric populations of closely related species of the genus *Littorina* (Neritrema)

Arina L. Maltseva[1], Marina A. Varfolomeeva[1], Roman V. Ayanka[1], Elizaveta R. Gafarova[1], Egor A. Repkin[1], Polina A. Pavlova[1], Alexei L. Shavarda[2,3], Natalia A. Mikhailova[1,4], Andrei I. Granovitch[1]

1 Department of Invertebrate Zoology, St. Petersburg State University, St. Petersburg, Russia
2 Department of Analytical Phytochemistry, Komarov Botanical Institute, St. Petersburg, Russia
3 Research Park, Centre for Molecular and Cell Technologies, St. Petersburg State University, St.-Petersburg, Russia
4 Centre of Cell Technologies, Institute of Cytology Russian Academy of Sciences, St. Petersburg, Russia

Appendix 2. Details of the analysis of microhabitat distribution.

Summary of the Ecological Preferences. Ecological preferences of the *Littorina* species in the sympatric populations can be outlined as follows. At least two species (*L. obtusata* and *L. saxatilis*) switch their preferences depending on intertidal level.

L. arcana occupies the upper part of the intertidal area, open sites with gravel substrate; it also expands down to the middle part in association with *F. vesiculosus* (personal observations), keeping itself mostly on the fucoid's surface.

L. compressa is strongly associated with the surface of *F. vesiculosus* both in the lower and upper littoral zone; rarely it can be found in open microhabitats.

L. fabalis is present exclusively in the lower littoral zone, often in association with *F. serratus*; nevertheless, it can be sporadically met on *A. nodosum* and *F. vesiculosis* (which are also present in the lower part of the intertidal area); in all the cases *L. fabalis* is present in the upper part of a fucoid canopy.

L. obtusata inhabits both upper and lower parts of the intertidal area in association with different fucoid macroalgae; in the lower part it occurs on *A. nodosum*, while in the upper part (in the absence of that fucoid species) switches to *F. vesiculosus*. It dwells mostly in the depth of a fucoid canopy.

L. saxatilis lives throughout the intertidal area as well. In both the upper and lower parts, it tends to occupy open stony (more frequently in the upper part) or gavel (usually in the lower part) microhabitats. Even when this species is associated with *F. vesicolosus* in both parts, it is present on gravel under the fucoid canopy (which indicates that this may be not a "true" association with the fucoid macroalgae, but a connection to a specific microbiotope under it).

A2 Table_1. The number of samples collected for analysis of microhabitat distribution of *Littorina* species grouped by collection site, shore level, and microhabitat.

Microhabitat	Saltstraumen		Varangerfjord	
	upper	lower	upper	lower
**Stone**	5			
**Gravel**		5	5	5
F. vesiculosus	5	5	5	5
F. serratus		5		5
A. nodosum		6		4

A2 Table_2. Effect of microhabitat on the distribution of *Littorina* species in the upper intertidal zone. Results of permutation test for partial distance-based redundancy analysis (dbRDA) after controlling for between-site differences. Bray-Curtis dissimilarities were calculated from the square root transformed species abundances in samples. Tests were performed with 9999 permutations.

Term	Df	SS	F	P
**Microhabitat**	2	1.01	5.94	0.0002
**Residual**	15	1.28		

A2 Table_3. Effect of microhabitat on the distribution of the *Littorina* species in the lower intertidal zone. Results of permutation test for partial distance-based redundancy analysis (dbRDA) after controlling for between-site differences. Bray Curtis dissimilarities were calculated from the square root transformed species abundances in samples. Tests were performed with 9999 permutations.

Term	Df	SS	F	P
**Microhabitat**	3	1.25	2.85	0.0015
**Residual**	34	4.98		

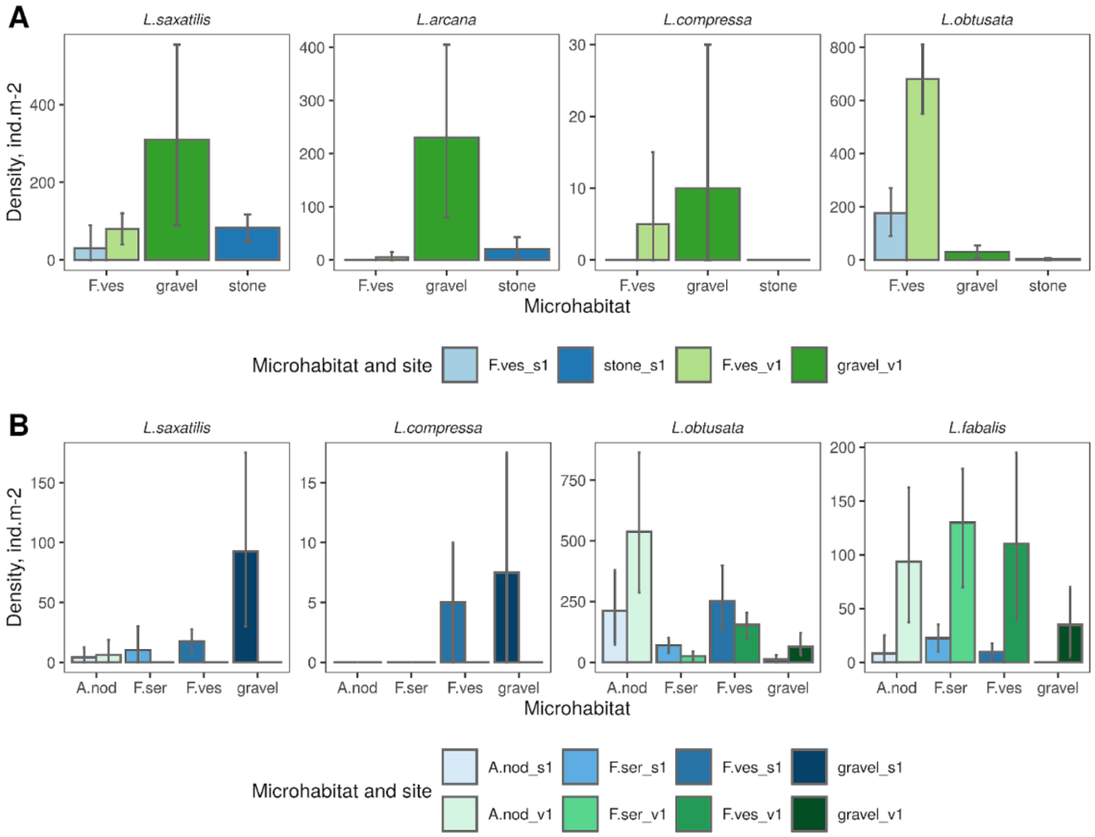

A2 Figure_1. Distribution of *Littorina* species in different microhabitats. A – in the upper part of the intertidal zone; B – in the lower part of the intertidal zone. Microhabitat types: F. ves – clumps of *Fucus vesiculosus*, gravel – open gravel, stone – open stone; A. nod – clumps of *Ascophyllum nodosum*; F. ser – clumps of *F. serratus*. Site: s1 - Saltstraumen; v1 - Varangerfjord. Mean densities (individuals m^{-2}) are shown with 95% confidence intervals obtained via bootstrap with 1000 iterations.

A2 Table_4. Effect of microhabitat and layer within the macroalgal canopy on the distribution of the *Littorina* species in the lower intertidal zone, in samples associated with fucoid macroalgae. Results of permutation tests for partial distance-based redundancy analysis (dbRDA) after controlling for between-site differences. The ordination model was

significant as a whole at $p = 0.0233$ in the permutational test. Bray-Curtis dissimilarities were calculated from the log-transformed species abundances in samples. Tests were performed with 9999 permutations.

Term	Df	SS	F	P
**Microhabitat**	2	2.34	8.35	0.0493
**Layer**	2	0.60	2.15	0.0233
**Residual**	73	10.21		

A2 Table_5. Effect of the layer within the macroalgal canopy and intertidal level on the distribution of the *Littorina* species within clumps of *F. vesiculosus*. Results of permutation tests for partial distance-based redundancy analysis (dbRDA) after controlling for between-site differences. The ordination model was significant as a whole at $p = 0.0001$ in the permutational test. Bray-Curtis dissimilarities were calculated from the square root transformed species abundances in samples. Tests were performed with 9999 permutations.

Term	Df	SS	F	P
**Level**	1	0.58	5.44	0.2275
**Layer**	2	0.97	4.54	0.0001
**Residual**	44	4.71		

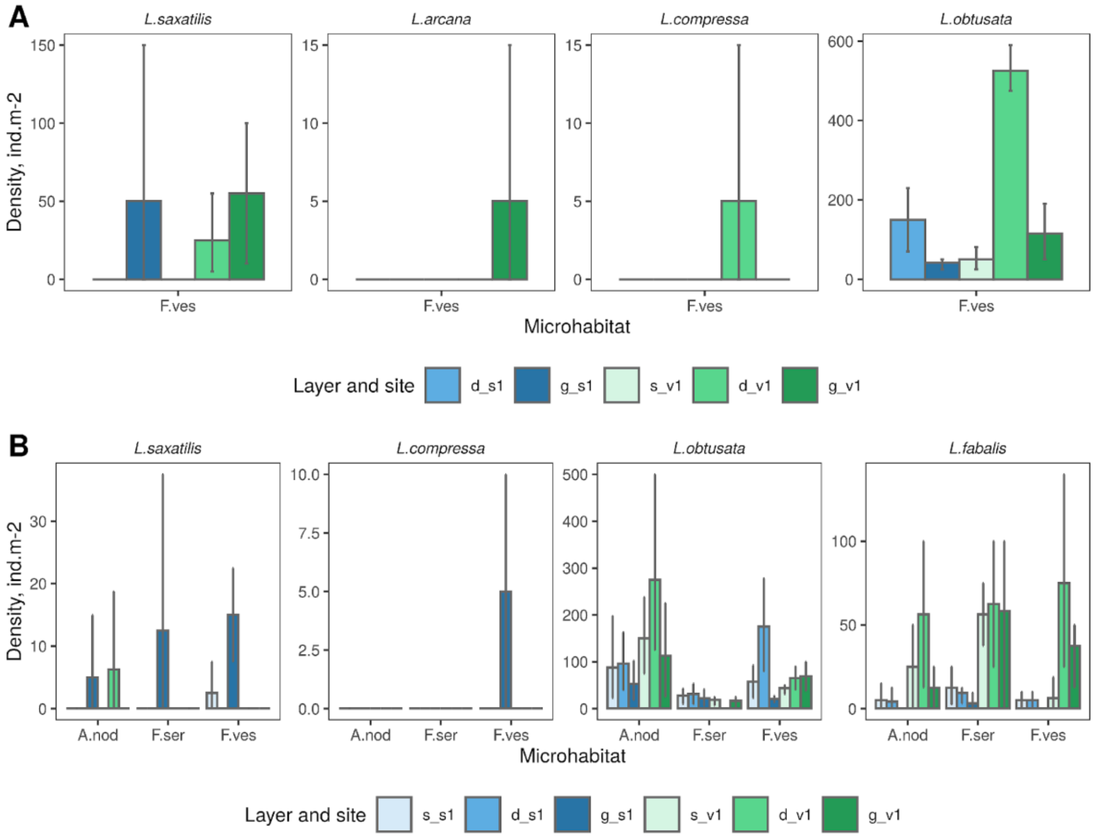

A2 Figure_2. Distribution of *Littorina* species in different layers of fucoid algae-related microhabitats. A – in the upper part of the intertidal zone in clumps of *Fucus vesiculosus*; B – in the lower part of the intertidal zone in clumps of *Ascophyllum nodosum* (A. nod), *F. serratus* (F. ser) and *F. vesiculosus* (F. ves). Layers and Site: s1 - Saltstraumen; v1 – Varangerfjord. Mean densities (individuals m^{-2}) are shown with 95% confidence intervals obtained via bootstrap with 1000 iterations.
