# Supplementary material for: Linking ecology, morphology, and metabolism: Niche differentiation in sympatric populations of closely related species of the genus Littorina (Neritrema)
Source: Ecol Evol. 2021 Jul 22;11(16):11134–54. doi: 10.1002/ece3.7901 (PMC8366845; doi:10.1002/ece3.7901)
Supplement: Supplementary file 3 — Appendix S3 [file ECE3-11-11134-s006.pdf]

Linking ecology, morphology and metabolism: niche differentiation in sympatric populations of closely related species of the genus *Littorina* (Neritrema)

Arina L. Maltseva[1], Marina A. Varfolomeeva[1], Roman V. Ayanka[1], Elizaveta R. Gafarova[1], Egor A. Repkin[1], Polina A. Pavlova[1], Alexei L. Shavarda[2,3], Natalia A. Mikhailova[1,4], Andrei I. Granovitch[1]

1 Department of Invertebrate Zoology, St. Petersburg State University, St. Petersburg, Russia
2 Department of Analytical Phytochemistry, Komarov Botanical Institute, St. Petersburg, Russia
3 Research Park, Centre for Molecular and Cell Technologies, St. Petersburg State University, St.-Petersburg, Russia
4 Centre of Cell Technologies, Institute of Cytology Russian Academy of Sciences, St. Petersburg, Russia

Appendix_3. Details of morphometric analysis.

A3 Table_1. The total number of *Littorina* snails collected for geometric morphometric analysis. Counts of healthy adult molluscs (no trematode infection, well developed reproductive system) are categorised by species, shore level, sex and collection site.

Species/subpopulation	Saltsraumen		Varangerfjord	
	females	males	females	males
***L. saxatilis*, upper level**	22	13	10	15
***L. saxatilis*, lower level**	27	16	17	6
L. arcana	3	5	18	6
L. compressa	15	7	15	10
***L. obtusata*, upper level**	20	12	11	13
***L. obtusata*, lower level**	24	23	10	15
L. fabalis	10	6	8	17

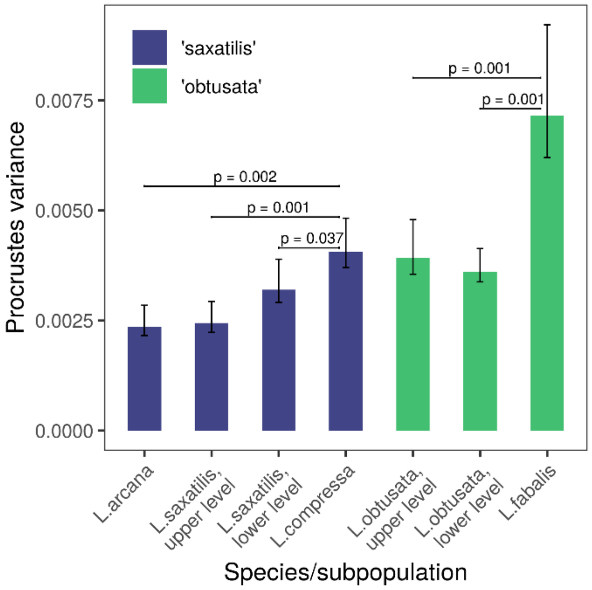

A3 Fig_1. Morphological disparity of five *Littorina* species and subpopulations. Among species and subpopulations studied, the highest values of the intraspecies morphological disparity were detected in the most low-dwelling species: L. fabalis, L. obtusata and L. compressa. On the contrary, the lowest degree of morphological disparity was revealed in most high-dwelling species: L. arcana and upper subpopulations of L. saxatilis. Disparity of shell shape was measured as Procrustes variance within species and their subpopulations (upper or lower intertidal level). Error bars are 95% bootstrap confidence limits with BCa correction computed using 999 iterations; arc – *L. arcana*, comp – *L. compressa*, fab – *L. fabalis*, obt – *L. obtusata*, sax – *L. saxatilis*.

A3 Table_2. Analysis of shape change in *Littorina* species. perMANOVA with randomised residual permutation procedure was performed on Procrustes distances among shapes. The model included the following predictors: log-transformed

centroid size, species/subpopulation (a categorical predictor with 7 levels for different species and upper- and lower-shore subpopulations of *L.saxatilis* and *L.obtusata*), collection site, and their interactions. Tests were performed with 999 permutations.

Term	Df	SS	MS	R^2	F	Z	P
**LogSize**	1	0.251	0.251	0.06	4.449	1.6	0.057
**Species/subpopulation**	6	1.573	0.262	0.35	6.034	3.0	0.002
**Site**	1	0.374	0.374	0.08	92.720	7.7	0.001
**LogSize : species/subpopulation**	6	0.141	0.023	0.03	2.132	2.0	0.025
**LogSize : site**	1	0.056	0.056	0.01	14.001	4.9	0.001
**Species/subpopulation : site**	6	0.261	0.043	0.06	10.771	10.0	0.001
**LogSize : species/subpopulation : site**	6	0.066	0.011	0.01	2.732	4.4	0.001
**Residuals**	346	1.396	0.004	0.31			
**Total**	373	4.469					

3 Table_3. Post hoc comparisons of morphological disparity in *Littorina* species. Comparisons were based on separate perMANOVA models for each species group (see below, Table 4, 5, Appendix_3). Between-site allometric differences had been removed (null model for comparisons included centroid size, site and their interaction); d — pairwise distances between shape variances; UCL (95%) — upper confidence limit; Z — standardised effect size. P-p-values.

Group of species	Contrast	d	UCL (95%)	Z	P
‘saxatilis’	***L. arcana* : *L. compressa***	0.002	0.001	4.3	0.002
	***L. arcana* : *L. saxatilis*, upper level**	<0.001	0.001	-1.0	0.851
	***L. arcana* : *L. saxatilis*, lower level**	0.001	0.001	1.6	0.082
	***L. compressa* : *L. saxatilis*, upper level**	0.002	0.001	4.9	0.001
	***L. compressa* : *L. saxatilis*, lower level**	0.001	0.001	2.2	0.037
	***L. saxatilis*, upper : lower level**	0.001	0.001	1.7	0.068
‘obtusata’	***L. fabalis* : *L. obtusata*, upper level**	0.003	0.002	5.3	0.001
	***L. fabalis* : *L. obtusata*, lower level**	0.002	0.001	6.4	0.001
	***L. obtusata*, upper : lower level**	<0.001	0.001	-0.5	0.638

A3_Table_4. Analysis of shape change within the ‘saxatilis’ group of *Littorina* species. perMANOVA with randomised residual permutation procedure was performed on Procrustes distances among shapes. The model included the following predictors: log-transformed centroid size, species/subpopulation (a categorical predictor with 4 levels: *L.arcana*, *L.compressa*, and upper- and lower-shore subpopulations of *L.saxatilis*), collection site, and their interactions. Tests were performed with 999 permutations.

Term	Df	SS	MS	R^2	F	Z	P
**LogSize**	1	0.04	0.041	0.04	3.26	1.3	0.098
**Species/subpopulation**	3	0.09	0.028	0.08	1.24	0.1	0.459
**Site**	1	0.13	0.127	0.12	38.45	6.4	0.001
**LogSize : species/subpopulation**	3	0.02	0.006	0.02	1.44	0.7	0.241
**LogSize : site**	1	0.01	0.012	0.01	3.78	2.6	0.001
**Species/subpopulation : site**	3	0.07	0.023	0.06	6.95	5.6	0.001
**LogSize : species/subpopulation : site**	3	0.01	0.004	0.01	1.35	1.1	0.119
**Residuals**	189	0.62	0.003	0.58			
**Total**	204	1.08					

A3_Table_5. Analysis of shape change within the ‘obtusata’ group of *Littorina* species. perMANOVA with randomised residual permutation procedure was performed on Procrustes distances among shapes. The model included the following predictors: log-transformed centroid size, species/subpopulation (a categorical predictor with 3 levels: *L. fabalis,* and

upper- and lower-shore subpopulations of *L. obtusata*), collection site, and their interactions. Tests were performed with 999 permutations.

Term	Df	SS	MS	R^2	F	Z	P
**LogSize**	1	0.27	0.267	0.15	3.98	1.3	0.095
**Species/subpopulation**	2	0.11	0.055	0.06	1.54	0.5	0.290
**Site**	1	0.34	0.336	0.19	68.30	7.1	0.001
**LogSize : species/subpopulation**	2	0.04	0.020	0.02	1.37	0.5	0.303
**LogSize : site**	1	0.07	0.067	0.04	13.68	4.9	0.001
**Species/subpopulation : site**	2	0.07	0.036	0.04	7.31	4.9	0.001
**LogSize : species/subpopulation : site**	2	0.03	0.015	0.02	3.00	2.9	0.002
**Residuals**	157	0.77	0.005	0.44			
**Total**	168	1.75					

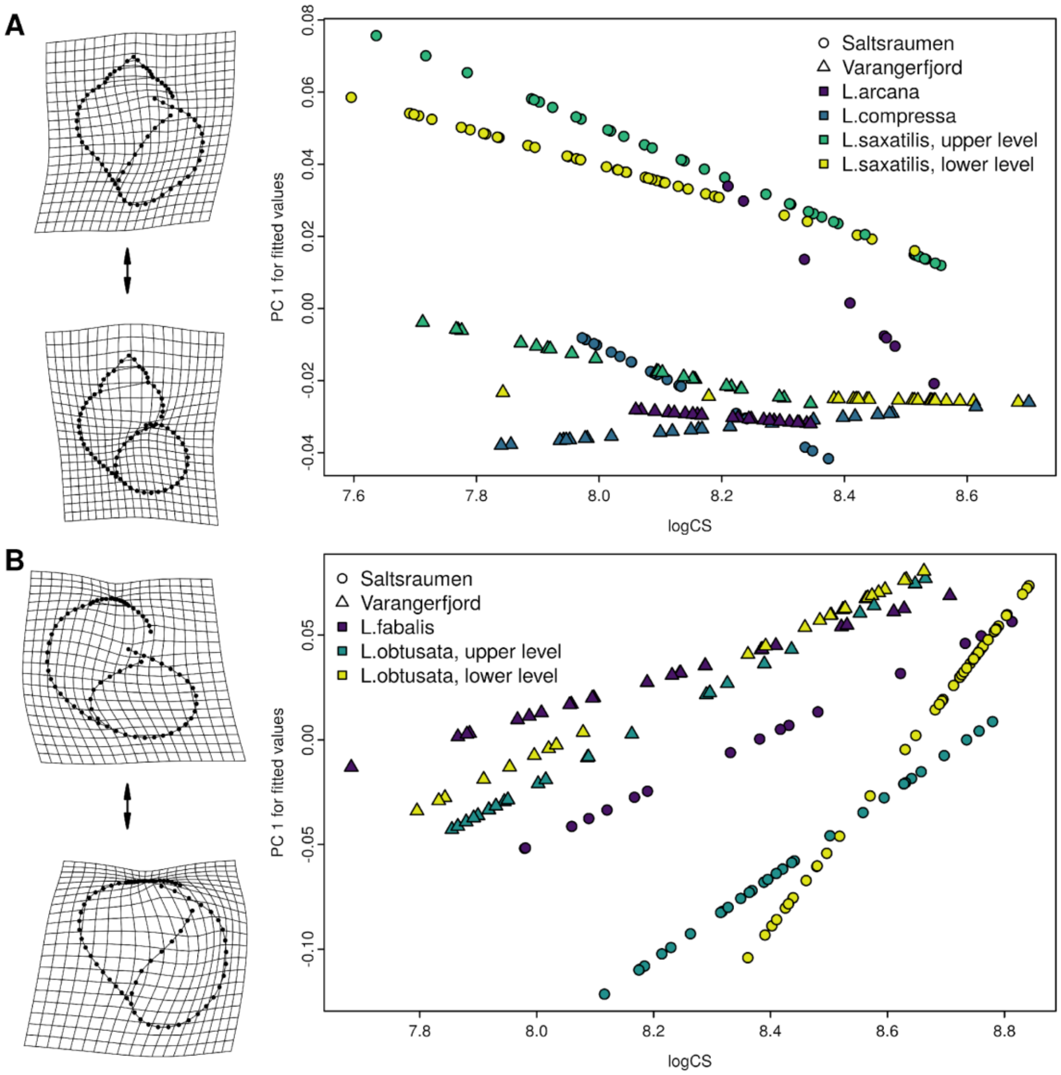

A3 Fig_2. Allometric trajectories of shell shape change in *Littorina* species and subpopulations. A – “saxatilis” group of species; B – “obtusata” group of species. The X-axis represents log-transformed centroid size. The y-axis is the first principal component of the predicted values of the multivariate regression of shape on size (see above, Table 4, 5, Appendix_3). Each line of dots is the predicted allometric trajectory for each species and subpopulation. Thin plate spline

transformation grids show the shape change from the mean shape of the corresponding species group to the highest (above) and the lowest (below) specimen on the Y-axis; shape changes were magnified by 1.5 for clarity (except for the lowest specimen on the Y-axis on the panel B, where the shape changes were magnified 1.2 times).

A3 Table_6. Analysis of shape change in *Littorina saxatilis*. perMANOVA with randomised residual permutation procedure was performed on Procrustes distances among shapes. The model was fitted with log-transformed centroid size, sex and intertidal level as fixed effects, the random effect of collection site, and interactions of these factors. Tests were performed with 999 permutations.

Term	Df	SS	MS	R^2	F	Z	P
**LogSize**	1	0.04	0.043	0.07	5.45	2.1	0.016
**Sex**	1	0.01	0.005	0.01	3.28	1.6	0.067
**Site**	1	0.08	0.081	0.14	26.57	6.2	0.001
**Level**	1	0.01	0.011	0.02	1.14	0.1	0.477
**LogSize:sex**	1	0.00	0.002	0.00	0.60	-0.7	0.773
**LogSize:site**	1	0.01	0.008	0.01	2.59	2.0	0.012
**LogSize:level**	1	0.01	0.008	0.01	2.71	1.2	0.116
**Sex:site**	1	0.00	0.002	0.00	0.55	-0.9	0.810
**Sex:level**	1	0.00	0.002	0.00	0.50	-1.0	0.848
**Site:level**	1	0.01	0.010	0.02	3.16	2.4	0.004
**LogSize:sex:site**	1	0.00	0.003	0.01	1.15	0.5	0.298
**LogSize:sex:level**	1	0.00	0.002	0.00	0.77	-0.3	0.624
**LogSize:site:level**	1	0.00	0.003	0.01	1.01	0.3	0.374
**Sex:site:level**	1	0.00	0.004	0.01	1.34	0.9	0.198
**LogSize:sex:site:level**	1	0.00	0.003	0.00	0.93	0.1	0.478
**Residuals**	110	0.33	0.003	0.57			
**Total**	125	0.58					

A3 Table_7. Analysis of shape change in *Littorina arcana*. perMANOVA with randomised residual permutation procedure was performed on Procrustes distances among shapes. The model was fitted with log-transformed centroid size and sex as fixed effects, the random effect of collection site, and interactions. Tests were performed with 999 permutations.

Term	Df	SS	MS	R^2	F	Z	P
**LogSize**	1	0.01	0.007	0.06	2.76	1.1	0.151
**Sex**	1	0.00	0.003	0.03	2.50	1.0	0.164
**Site**	1	0.01	0.011	0.09	3.84	2.6	0.001
**LogSize:sex**	1	0.00	0.001	0.01	0.54	-0.8	0.784
**LogSize:site**	1	0.00	0.003	0.02	0.92	0.2	0.443
**Sex:site**	1	0.00	0.001	0.01	0.47	-1.0	0.836
**LogSize:sex:site**	1	0.00	0.003	0.02	0.95	0.2	0.432
**Residuals**	24	0.07	0.003	0.58			
**Total**	31	0.11					

A3 Table_8. Analysis of shape change in *Littorina compressa*. perMANOVA with randomised residual permutation procedure was performed on Procrustes distances among shapes. The model was fitted with log-transformed centroid size and sex as fixed effects, the random effect of collection site, and interactions. Tests were performed with 999 permutations.

Term	Df	SS	MS	R^2	F	Z	P
**LogSize**	1	0.00	0.003	0.01	0.73	-0.4	0.660
**Sex**	1	0.03	0.026	0.09	1.76	0.5	0.310
**Site**	1	0.07	0.070	0.25	19.64	4.8	0.001
**LogSize:sex**	1	0.01	0.006	0.02	1.45	0.3	0.368
**LogSize:site**	1	0.00	0.004	0.01	0.99	0.3	0.406
**Sex:site**	1	0.01	0.015	0.05	4.19	2.7	0.003

**LogSize:sex:site**	1	0.00	0.004	0.02	1.20	0.6	0.278
**Residuals**	39	0.14	0.004	0.50			
**Total**	46	0.28					

A3 Table_9. Analysis of shape change in *Littorina obtusata*. perMANOVA with randomised residual permutation procedure was performed on Procrustes distances among shapes. The model was fitted with log-transformed centroid size, sex and intertidal level as fixed effects, the random effect of collection site, and interactions. Tests were performed with 999 permutations.

Term	Df	SS	MS	R^2	F	Z	P
**LogSize**	1	0.25	0.251	0.21	3.65	1.1	0.147
**Sex**	1	0.00	0.003	0.00	0.95	-0.1	0.557
**Site**	1	0.25	0.254	0.21	64.11	6.5	0.001
**Level**	1	0.03	0.033	0.03	2.30	1.0	0.167
**LogSize:sex**	1	0.00	0.002	0.00	0.16	-2.4	0.993
**LogSize:site**	1	0.07	0.069	0.06	17.40	5.1	0.001
**LogSize:level**	1	0.02	0.021	0.02	1.45	0.4	0.351
**Sex:site**	1	0.00	0.003	0.00	0.78	-0.2	0.562
**Sex:level**	1	0.00	0.004	0.00	0.61	-0.6	0.754
**Site:level**	1	0.01	0.014	0.01	3.58	2.5	0.007
**LogSize:sex:site**	1	0.01	0.010	0.01	2.47	1.9	0.032
**LogSize:sex:level**	1	0.01	0.005	0.00	0.77	-0.3	0.621
**LogSize:site:level**	1	0.01	0.014	0.01	3.59	2.6	0.005
**Sex:site:level**	1	0.01	0.007	0.01	1.70	1.2	0.108
**LogSize:sex:site:level**	1	0.01	0.007	0.01	1.73	1.3	0.107
**Residuals**	112	0.44	0.004	0.37			
**Total**	127	1.20					

A3 Table_10. Analysis of shape change in *Littorina fabalis*. perMANOVA with randomised residual permutation procedure was performed on Procrustes distances among shapes. The model was fitted with log-transformed centroid size and sex as fixed effects, the random effect of collection site, and interactions. Tests were performed with 999 permutations.

Term	Df	SS	MS	R^2	F	Z	P
**LogSize**	1	0.02	0.024	0.05	5.09	1.7	0.044
**Sex**	1	0.01	0.006	0.01	1.30	0.2	0.429
**Site**	1	0.12	0.119	0.25	15.14	4.6	0.001
**LogSize:sex**	1	0.01	0.009	0.02	0.72	-0.4	0.662
**LogSize:site**	1	0.00	0.005	0.01	0.61	-0.5	0.661
**Sex:site**	1	0.00	0.005	0.01	0.58	-0.6	0.713
**LogSize:sex:site**	1	0.01	0.013	0.03	1.63	1.1	0.143
**Residuals**	33	0.26	0.008	0.55			
**Total**	40	0.47					
