# Supplementary material for: Linking ecology, morphology, and metabolism: Niche differentiation in sympatric populations of closely related species of the genus Littorina (Neritrema)
Source: Ecol Evol. 2021 Jul 22;11(16):11134–54. doi: 10.1002/ece3.7901 (PMC8366845; doi:10.1002/ece3.7901)
Supplement: Supplementary file 4 — Appendix S4 [file ECE3-11-11134-s005.pdf]

Linking ecology, morphology and metabolism: niche differentiation in sympatric populations of closely related species of the genus *Littorina* (Neritrema)

Arina L. Maltseva[1], Marina A. Varfolomeeva[1], Roman V. Ayanka[1], Elizaveta R. Gafarova[1], Egor A. Repkin[1], Polina A. Pavlova[1], Alexei L. Shavarda[2,3], Natalia A. Mikhailova[143], Andrei I. Granovitch[1]

1 Department of Invertebrate Zoology, St. Petersburg State University, St. Petersburg, Russia
2 Department of Analytical Phytochemistry, Komarov Botanical Institute, St. Petersburg, Russia
3 Research Park, Centre for Molecular and Cell Technologies, St. Petersburg State University, St.-Petersburg, Russia
4 Centre of Cell Technologies, Institute of Cytology Russian Academy of Sciences, St. Petersburg, Russia

Appendix 4. Details of gas chromatography mass-spectrometry analysis.

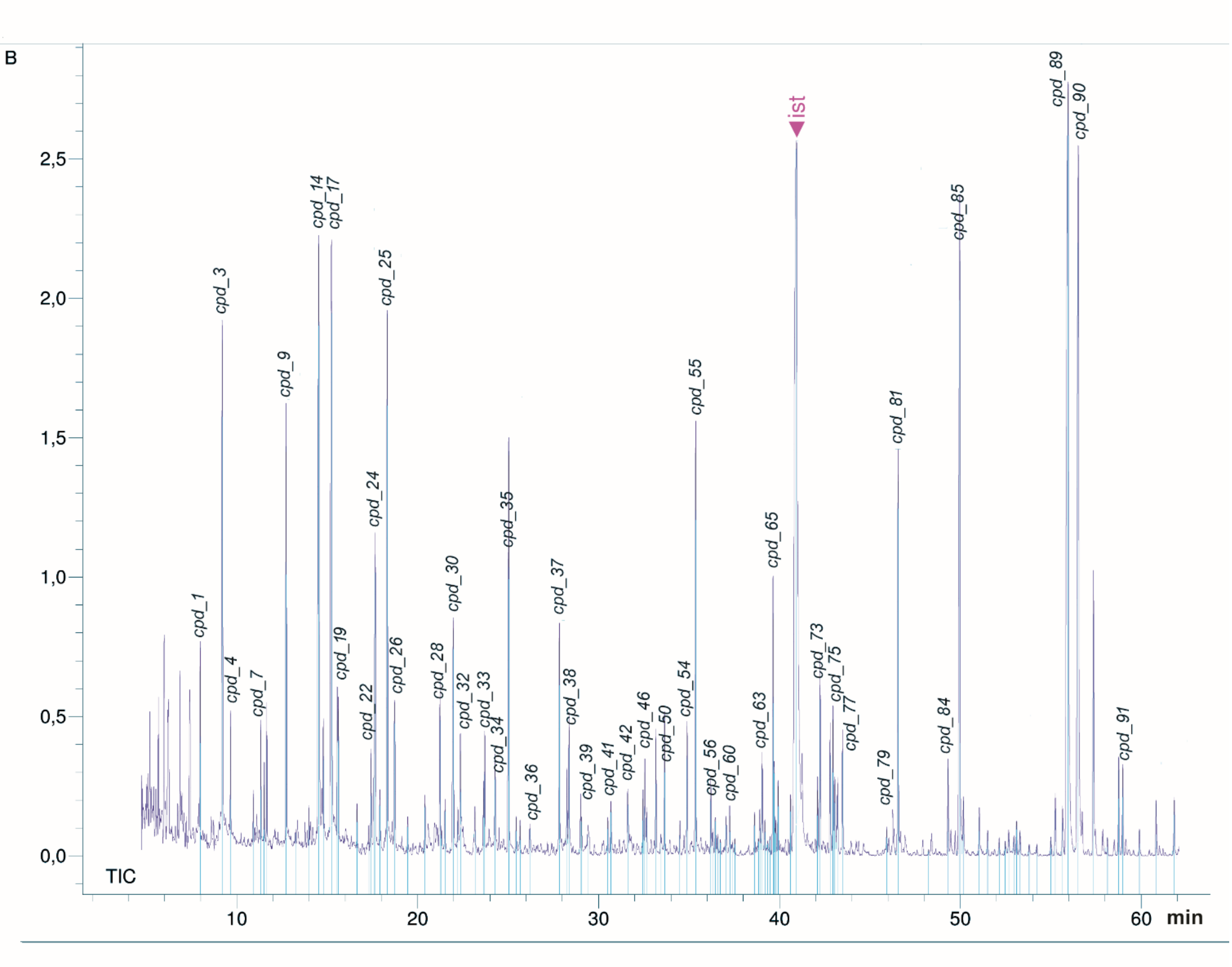

A4 Fig_1. TIC-chromatogram of GC-MS/MS analysis. The gas chromatography mass spectrometry analysis (GC-MS) was carried out on gas chromatograph Agilent 7890 with time-of-flight mass spectrometer Leco Pegasus 4D GCxGC-TOF MS; the carrier gas was helium, the column was Zorbax DB5 (length 30 m, inner diameter 0.25 mm, film thickness 0.25 µm). The initial temperature was 70 °C, final temperature was 320 °C, the gradient was 6 °C per minute. The injector temperature was 250 °C. The scanning frequency was ten spectra per second in a range of masses 50-800 Da. Metabolites identification was made using fragmented spectra NIST (National Institute of Standards and Technology) database and standard chromatograms of single amino acids, monosaccharides, fatty acids manually and their mixtures. Quantitation of metabolites was performed by peak total ion current (TIC) on the chromatogram.

A4 Table_1. Metabolites identification. Compounds were numbered according to the order of elution; unk[principal ion mass] code for compounds not identified based on available NIST database and standards, TMS code for trimethylsilyl groups number, P code for monosaccharide in pyranose form, F code for monosaccharide in furanose form, MAG code for monoacylglycerol.

**Compound**	**Identification**	**Retention time**	**Retention index**
**cpd_1**	lactate	7,98	1060,0
**cpd_2**	unk[70,174]	8,73	1088,2
**cpd_3**	alanine	9,20	1106,0
**cpd_4**	glycine	9,66	1123,0
**cpd_5**	unk[267]	10,77	1164,9
**cpd_6**	pipecolate	10,91	1170,4
**cpd_7**	methyl phosphate	11,33	1185,8
**cpd_8**	unk[237]	11,52	1193,2
**cpd_9**	valine	12,73	1238,8
**cpd_10**	unk[55]	13,12	1253,3
**cpd_11**	methyl glycerate	13,25	1258,2
**cpd_12**	caprolactam	14,00	1286,4
**cpd_13**	unk[95,155]	14,26	1296,2
**cpd_14**	phosphate	14,53	1306,4
**cpd_15**	unk[158,207]	14,61	1309,4
**cpd_16**	glycerol	14,78	1315,8
**cpd_17**	proline	15,24	1333,2
**cpd_18**	unk[158]	15,31	1335,7
**cpd_19**	picolinate	15,55	1344,9
**cpd_20**	succinate	15,62	1347,2
**cpd_21**	unk[73]	16,64	1385,9
**cpd_22**	serine	17,42	1415,2
**cpd_23**	asparagine	17,66	1424,2
**cpd_24**	unk[239]	17,91	1433,5
**cpd_25**	threonine	18,32	1449,0
**cpd_26**	N-methylglutamate	18,72	1464,0
**cpd_27**	unk[71]	20,40	1527,3
**cpd_28**	malate	21,31	1561,6
**cpd_29**	unk[156]	21,91	1584,0
**cpd_30**	pyroglutamate+methionine	21,97	1586,3
**cpd_31**	aspartate	22,22	1595,8
**cpd_32**	2-aminoethylphosphonate [3TMS]	22,36	1600,8
**cpd_33**	F-phospate	23,71	1651,7
**cpd_34**	hypotaurine	24,28	1673,2
**cpd_35**	phenylalanine	25,03	1701,5
**cpd_36**	taurine	26,20	1745,3
**cpd_37**	aC11:0, undecanoic acid	28,25	1822,6
**cpd_38**	2-aminoethylphosphoric acid	28,37	1827,0
**cpd_39**	phosphorylethanolamine	29,40	1866,0
**cpd_40**	P[204]	30,51	1907,5
**cpd_41**	aC14:0, myristic acid	30,68	1913,9
**cpd_42**	tyrosine[2TMS]	31,60	1948,7
**cpd_43**	unk[55]	31,89	1959,5
**cpd_44**	P[204]	32,21	1971,5

**cpd_45**	Me aC16:0, palmitic acid methyl ester	32,46	1980,8
**cpd_46**	P, glucose_1	32,56	1984,5
**cpd_47**	unk[133]	32,65	1988,0
**cpd_48**	unk[73]	32,77	1992,6
**cpd_49**	tyrosine [3TMS]	33,17	2007,6
**cpd_50**	hexitol	33,26	2011,0
**cpd_51**	aC14:0, myristic acid	33,44	2017,8
**cpd_52**	unk[87]	33,53	2021,2
**cpd_53**	pantothenic acid	33,64	2025,5
**cpd_54**	P, glucose_2	34,89	2072,5
**cpd_55**	aC16:0, palmitic acid	35,36	2090,2
**cpd_56**	F	36,20	2121,8
**cpd_57**	Me aC18:2, linoleic acid methyl ester	36,30	2125,2
**cpd_58**	Me aC18:3, α-linolenic acid methyl ester	36,45	2131,1
**cpd_59**	aC17:0, margaric acid	36,73	2141,6
**cpd_60**	unk[231] + Me aC18:0, stearic acid	37,04	2153,3
**cpd_61**	unk[147]	37,53	2171,5
**cpd_62**	tryptophane [4TMS]	38,83	2220,6
**cpd_63**	aC18:1a, oleic acid	39,04	2228,6
**cpd_64**	aC18:1b, elaidic acid	39,19	2234,1
**cpd_65**	aC18:0	39,64	2251,0
**cpd_66**	glutamate	39,70	2253,3
**cpd_67**	Me aC20:4, arachidonic acid methyl ester	39,79	2256,7
**cpd_68**	Me aC20:5, eicosapentaenoic acid methyl ester	39,92	2261,6
**cpd_69**	Me aC20:2, eicosadienoic acid methyl ester	40,61	2287,5
**cpd_70**	unk[87]	40,75	2292,8
**cpd_71**	Ist = C23 standard	40,94	2300,0
**cpd_72**	aC20:4, arachidonic acid	42,10	2367,9
**cpd_73**	aC20:5, eicosapentaenoic acid	42,24	2376,1
**cpd_74**	MAG[0:0\16:0\0:0], 2-monopalmitylglycerol	42,80	2408,5
**cpd_75**	aC20:1-1, paullinic acid	42,96	2417,8
**cpd_76**	aC20:1-b, gondoic acid	43,05	2423,4
**cpd_77**	MAG[16:0\0:0\0:0], 1-monopalmitylglycerol	43,48	2448,1
**cpd_78**	unk[149]	44,39	2501,5
**cpd_79**	MAG[0:0\16:0\0:0], 2-monopalmitylglycerol	45,93	2591,3
**cpd_80**	unk	46,07	2599,6
**cpd_81**	MAG[16:0\0:0\0:0], 1-monopalmitylglycerol	46,55	2627,7
**cpd_82**	Adenosine	47,55	2686,1
**cpd_83**	Sucrose	48,17	2722,2
**cpd_84**	MAG[0:0\18:0\0:0] (2TMS), 2-monostearylglycerol	49,32	2789,3
**cpd_85**	MAG[18:0\0:0\0:0], 1-monostearylglycerol	49,96	2827,1
**cpd_86**	unk[340]	52,68	2985,8
**cpd_87**	unk[456]	55,25	3135,6
**cpd_88**	a-tocopherol	55,65	3159,2
**cpd_89**	cholesterol	55,95	3177,0
**cpd_90**	desmosterol	56,51	3207,9
**cpd_91**	stigmasterol	58,75	3332,5
