# Supplementary material for: Linking ecology, morphology, and metabolism: Niche differentiation in sympatric populations of closely related species of the genus Littorina (Neritrema)
Source: Ecol Evol. 2021 Jul 22;11(16):11134–54. doi: 10.1002/ece3.7901 (PMC8366845; doi:10.1002/ece3.7901)
Supplement: Supplementary file 5 — Appendix S5 [file ECE3-11-11134-s001.pdf]

Linking ecology, morphology and metabolism: niche differentiation in sympatric populations of closely related species of the genus *Littorina* (Neritrema)

Arina L. Maltseva[1], Marina A. Varfolomeeva[1], Roman V. Ayanka[1], Elizaveta R. Gafarova[1], Egor A. Repkin[1], Polina A. Pavlova[1], Alexei L. Shavarda[2,3], Natalia A. Mikhailova[1,4], Andrei I. Granovitch[1]

1 Department of Invertebrate Zoology, St. Petersburg State University, St. Petersburg, Russia
2 Department of Analytical Phytochemistry, Komarov Botanical Institute, St. Petersburg, Russia
3 Research Park, Centre for Molecular and Cell Technologies, St. Petersburg State University, St.-Petersburg, Russia
4 Centre of Cell Technologies, Institute of Cytology Russian Academy of Sciences, St. Petersburg, Russia

Appendix_5. Details on comparative interspecies metabolomic analysis.

A5 Table_1. Comparison of metabolomes of littorines. Results of two-way perMANOVA based on a matrix of Euclidean distances among samples (log-transformed weight- and quantile normalised abundances). The model included species, collection site, and their interaction as predictors. Tests were performed with 9999 permutations.

	Df	SS	MS	F	R^2	P
**Species**	4	53854	13463.4	5.25	0.18	0.0001
**Site**	1	11646	11640.8	4.54	0.04	0.0001
**Species:Site**	4	16212	4053.0	1.58	0.06	0.0032
**Residuals**	83	213001	2566.3		0.72	
**Total**	92	294708			1.00	

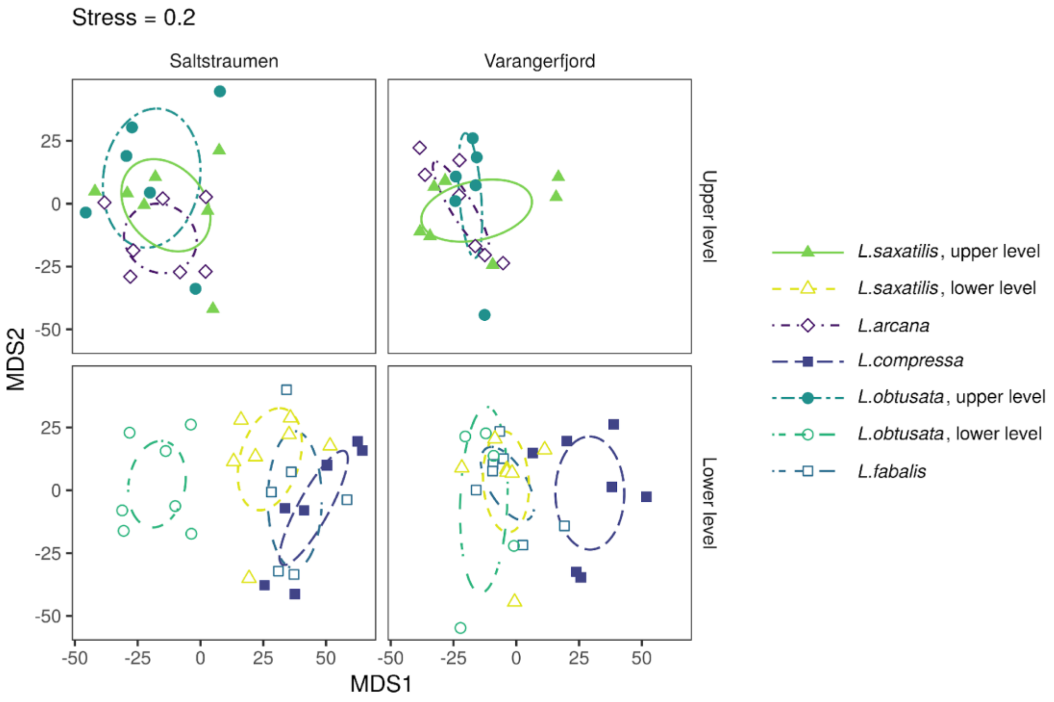

A5 Fig_1. nMDS ordination of individual metabolomes of five *Littorina* species grouped by intertidal level, species, and collection site. The metabolomes of inhabitants of the upper intertidal level (three species: *L. arcana*, *L. obtusata* and *L. saxatilis*) were more similar than those of the lower level (four species: *L. compressa*, *L. fabalis*, *L. obtusata* and *L. saxatilis*) in both sites. The interaction between “species/subpopulation” and “site” factors was significant due to more pronounced differences between intertidal levels in Saltstraumen, where the coast was more exposed to wave action.
