# Supplementary material for: Linking ecology, morphology, and metabolism: Niche differentiation in sympatric populations of closely related species of the genus Littorina (Neritrema)
Source: Ecol Evol. 2021 Jul 22;11(16):11134–54. doi: 10.1002/ece3.7901 (PMC8366845; doi:10.1002/ece3.7901)
Supplement: Supplementary file 6 — Appendix S6 [file ECE3-11-11134-s004.pdf]

Linking ecology, morphology and metabolism: niche differentiation in sympatric populations of closely related species of the genus *Littorina* (Neritrema)

Arina L. Maltseva[1], Marina A. Varfolomeeva[1], Roman V. Ayanka[1], Elizaveta R. Gafarova[1], Egor A. Repkin[1], Polina A. Pavlova[1], Alexei L. Shavarda[2,3], Natalia A. Mikhailova[1,4], Andrei I. Granovitch[1]

1 Department of Invertebrate Zoology, St. Petersburg State University, St. Petersburg, Russia
2 Department of Analytical Phytochemistry, Komarov Botanical Institute, St. Petersburg, Russia
3 Research Park, Centre for Molecular and Cell Technologies, St. Petersburg State University, St.-Petersburg, Russia
4 Centre of Cell Technologies, Institute of Cytology Russian Academy of Sciences, St. Petersburg, Russia

Supplement_6. Analysis of the level- and species-distinguishing metabolites

Among the metabolic compounds differentiating all five *Littorina* (*Neritrema*) species, there are adenosine, diverse saturated and unsaturated long chain fatty acids and their derivatives have been identified (A6 Tables_1-3). Adenosine is known to be an inhibitory neurotransmitter (when extracellular) in both vertebrates and invertebrates. Being a breakdown product of ATP, it plays a critical role in tissue tolerance to hypoxia (Nilsson & Lutz, 1992; Pek & Lutz, 1997; Reipschlager et al., 1997; Rebshow et al., 2002). This compound was most abundant in *L. obtusata* and in the lower fraction of *L. saxatilis*, probably participating in regulation of withstanding partial anaerobiosis during low tide (see also below).

Another class of differential metabolites includes fatty acids, their methyl esters and monoacylglycerols. All these compounds can be used as substrates for synthesis of membrane phospholipids, and their varying abundance may reflect a variation in the content of membrane lipids due to differential temperature regime, which is a well-known mechanism of temperature and osmotic adaptation in various organisms (Soudant *et al*., 1998; Guerzoni *et al*., 2001; Tchernov *et al*., 2004). This is also true for cholesterol, identified as an abundant metabolic marker in *L. obtusata* (Crockett, 1998). In addition, unsaturated fatty acids were described as structural units of lipochrome, a carotenoid pigment, stored in so-called cytosomes. Lipochrome can function as an endogenous electron acceptor under short-term an- / hypoxic conditions, allowing it to preserve the full functionality of mitochondria. This mechanism can act quickly and thus operates in the most important integral organs, such as neural ganglia or pericardium (Zs.-Nagy, 1977; Zavras & James, 1979; De Zwaan, 1983). Accordingly, nervous ganglia of littorines are yellow-pigmented (our personal observations). In general, marine gastropods (and some other marine fish and invertebrates) are prone to accumulation of various sorts of polyunsaturated fatty acids (Joseph, 1989; Saito & Aono. 2014). The revealed diversity of their relative content among the *Neritrema* species may reflect both the individuality of their metabolism and the specificity of their diet, which, in turn, is associated with the preferred microbiotope occupied.

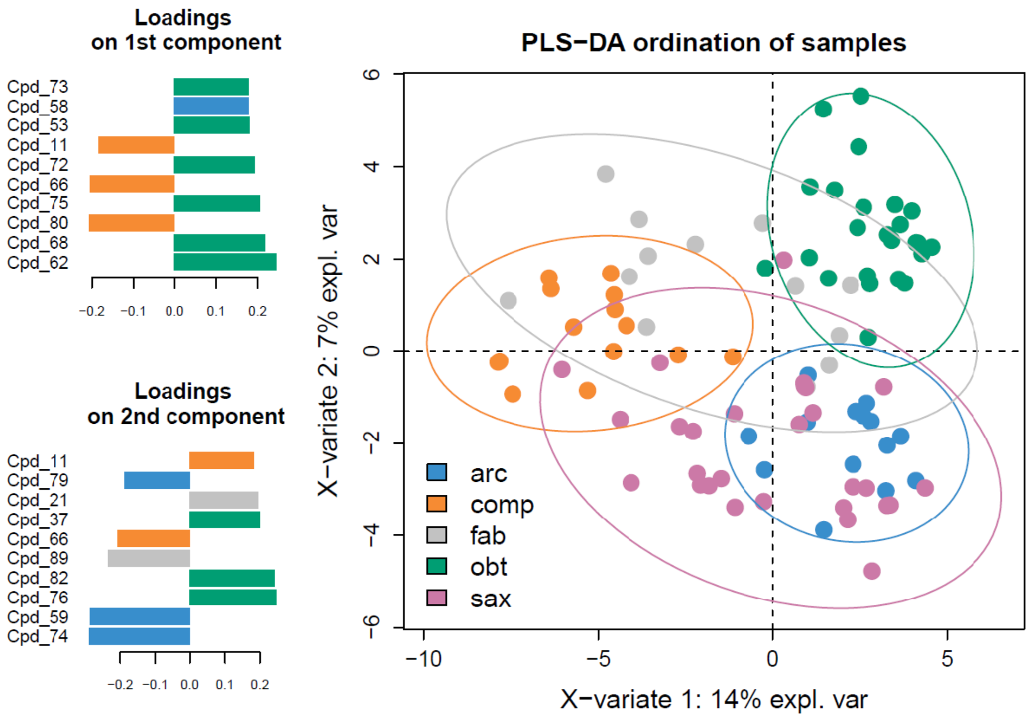

A6 Fig_1. PLS-DA of metabolomes by species. PLS-DA was applied to determine metabolites most effectively distinguishing *Littorina* species studied. Compounds were ranked based on their importance for differentiation of species according to loadings on the first two PLS-DA components. Loadings of the first ten compounds on each component are plotted; the colour of bars corresponds to the group with the highest mean abundance of the compound. The names of the compounds are listed below (see A6 Table 1, Appendix_6). The complete results of the identification of metabolites are in Appendix_4. arc – *L. arcana*, comp – *L. compressa*, fab – *L. fabalis*, obt – *L. obtusata*, sax – *L. saxatilis*.

A6 Table_1. Species-distinguishing metabolites. The key metabolites important for distinguishing five *Littorina* species according to PLS-DA (with the highest loadings on the first two components) are grouped by species in which their mean abundance is the highest (see above, A6 Fig_1, and Appendix_4). *L. saxatilis* occupied an intermediate position both in comparative interspecies PLS-DA (A6 Fig_1) and nMDS ordination (Fig_7). Consequently, among the species-distinguishing metabolites, there were no compounds with the highest mean abundance in *L. saxatilis*.

species	compound	function
L. arcana	Me aC18:3, α-linolenic acid methyl ester<br>aC17:0, margaric acid<br>Cpd_74, 79 MAG[0:0\16:0\0:0], 2-monopalmitylglycerol	lipid metabolism
L. compressa	Cpd_11, methyl glycerate	glycerate: an intermediate of glycolysis (modified during sample preparation)
	Cpd_66, glutamate	free amino acid: protein synthesis, osmoregulation
	Cpd_80, unknown	-
L. fabalis	Cpd_21, unknown	-
	Cpd_89, cholesterol	polycyclic alcohol: membrane regulation, steroids synthesis
L. obtusata	Cpd_53, pantothenic acid	vitamin B5: cofactor
	Cpd_62, tryptophane	free amino acid: protein synthesis, osmoregulation
	Cpd_37, aC11:0, undecanoic acid<br>Cpd_68, Me aC20:5, eicosapentaenoic acid methyl ester<br>Cpd_72, aC20:4, arachidonic acid<br>Cpd_73, aC20:5, eicosapentaenoic acid<br>Cpd_75, aC20:1-1, paullinic acid<br>Cpd_76, aC20:1-b, gondoic acid	lipid metabolism
	Cpd_82, adenosine	nucleoside

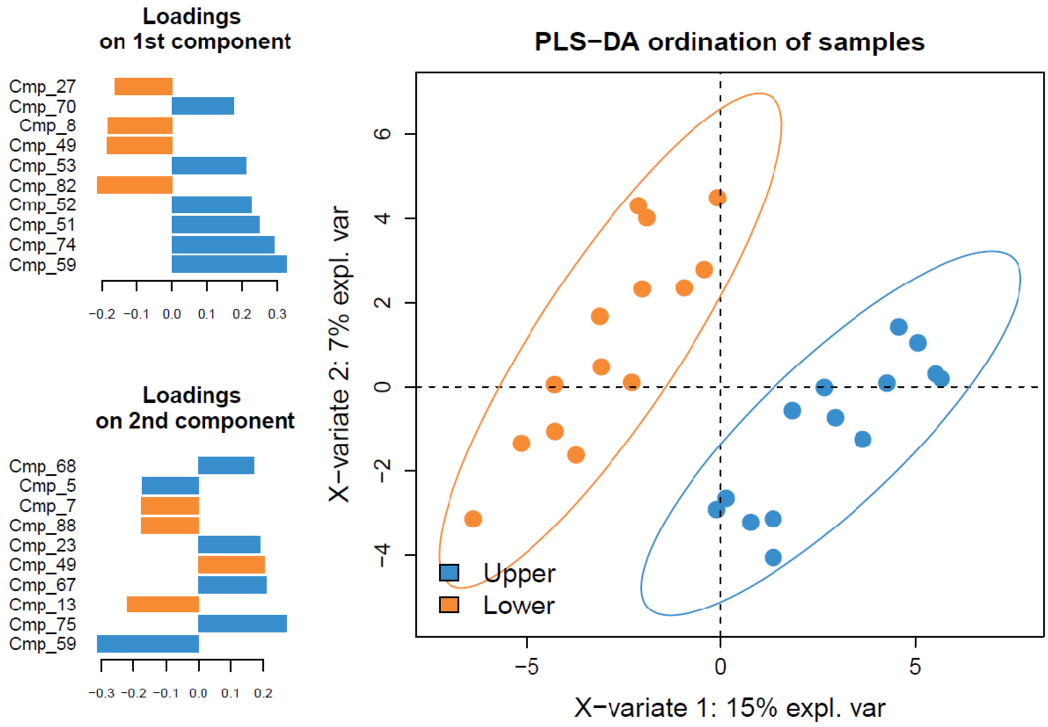

A6 Fig_2. PLS-DA of metabolomes of *L. saxatilis* by intertidal level. Key metabolites distinguishing subpopulations of *L. saxatilis* from different intertidal levels were identified using PLS-DA. Compounds are ranked based on their importance for differentiation of species according to loadings on the first two PLS-DA components. Loadings of the first ten compounds on each component are plotted; the colour of bars corresponds to the group with the highest mean abundance of the compound. The names of the compounds are listed below (A6 Table_2 and Appendix_4).

A6 Table_2. Metabolites of *L. saxatilis*, distinguishing subpopulation from different intertidal levels. The metabolites with the highest loadings on the first two components in PLS-DA are grouped by subpopulation in which their mean abundance is the highest (see above, A6 Fig_2 and Appendix_4).

level	compound	function
upper	Cpd_5, unknown	-
	Cpd_52, unknown	-
	Cpd_70, unknown	-
	Cpd_23, asparagine	free amino acid: protein synthesis, osmoregulation
	Cpd_53, pantothenic acid	vitamin B5: cofactor
	Cpd_51, aC14:0, myristic acid<br>Cpd_59, aC17:0, margaric acid<br>Cpd_67, Me aC20:4, arachidonic acid methyl ester<br>Cpd_68, Me aC20:5, eicosapentaenoic acid methyl ester<br>Cpd_74, MAG[0:0\16:0\0:0], 2-monopalmitylglycerol<br>Cpd_75, aC20:1-1, paullinic acid	lipid metabolism
lower	Cpd_8, unknown	-
	Cpd_13, unknown	-
	Cpd_27, unknown	-
	Cpd_7, methyl phosphate	-
	Cpd_49, tyrosine	free amino acid: protein synthesis, osmoregulation
	Cpd_82, adenosine	nucleoside
	Cpd_88, a-tocopherol	vitamin E: cofactor

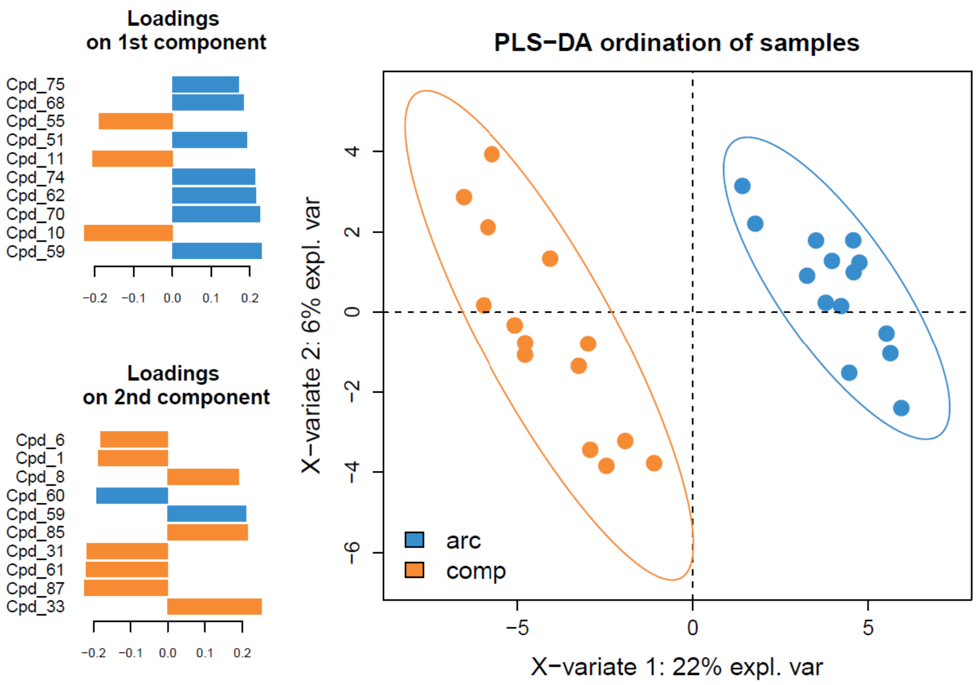

A6 Fig_3. PLS-DA of metabolomes of *L. arcana* and *L. compressa*. Molluscs of these species predominantly occupy different intertidal levels *(L. arcana* dwells in the upper level, while *L. compressa* is a lower shore species). Metabolomes of *L. arcana* and *L. compressa* partially overlapped the ones of *L. saxatilis* from upper and lower subpopulations correspondingly. We determined key metabolites distinguishing *L. arcana* and *L. compressa* using PLS-DA to identify common patterns in metabolome variation with intertidal level. Compounds are ranked based on their importance for differentiation of species according to loadings on the first two PLS-DA components. Loadings of the first ten compounds on each component are plotted; the colour of bars corresponds to the group with the highest mean abundance of the compound. The names of the compounds are listed below (A6 Table_3 and Appendix_4). arc – *L. arcana*, comp – *L. compressa*.

A6 Table_3. Metabolites, distinguishing *L. arcana* and *L. compressa*. The metabolites with the highest loadings on the first two components in PLS-DA are grouped by species in which their mean abundance is the highest (see above, A6 Fig_3 and Appendix_4).

species	compound	function
L. arcana	Cpd_51, aC14:0, myristic acid<br>Cpd_59, aC17:0, margaric acid<br>Cpd_68, Me aC20:5, eicosapentaenoic acid methyl ester<br>Cpd_74, MAG[0:0\16:0\0:0], 2-monopalmitylglycerol<br>Cpd_75, aC20:1-1, paullinic acid<br>Cpd_60, unk[231] + Me aC18:0, stearic acid	lipid metabolism
	Cpd_62, tryptophane	free amino acid: protein synthesis, osmoregulation
	Cpd_70, unknown	-
L. compressa	Cpd_8, unknown	-
	Cpd_10, unknown	-
	Cpd_61, unknown	-
	Cpd_6, pipecolate	-
	Cpd_11, methyl glycerate	glycerate: an intermediate of glycolysis (modified during sample preparation)
	Cpd_31, aspartate	free amino acid: protein synthesis, osmoregulation
	Cpd_33, monosaccharide-phosphate	sugar metabolism
	Cpd_55, aC16:0, palmitic acid<br>Cpd_81, MAG[16:0\0:0\0:0], 1-monopalmitylglycerol<br>Cpd_85, MAG[18:0\0:0\0:0], 1-monostearylglycerol	lipid metabolism

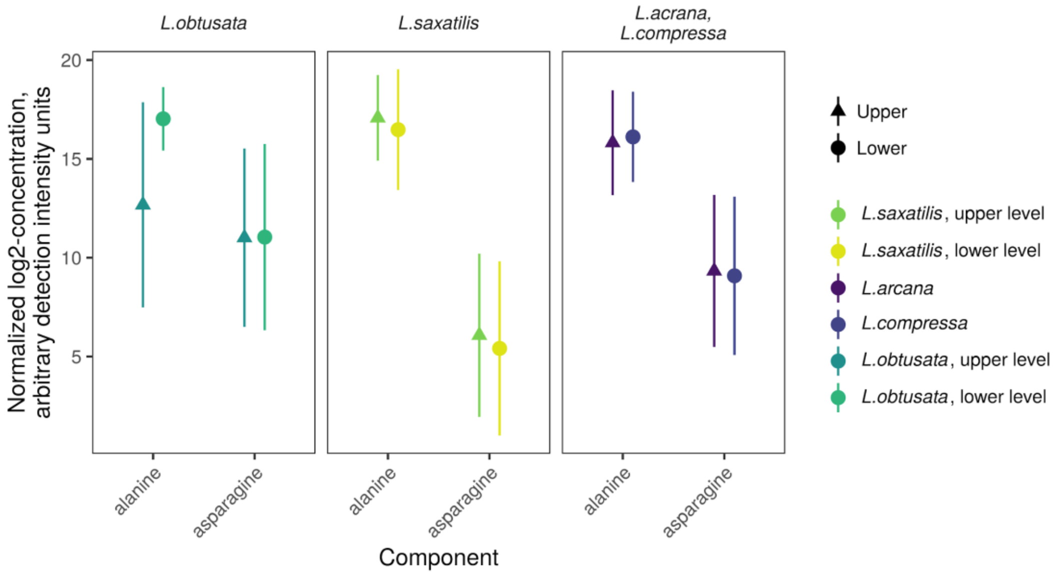

A6 Fig_4. Comparison of abundances of the amino acids involved in anaerobic metabolism in *Littorina* snails from different intertidal levels. Concentrations are in arbitrary detection intensity units. Means and 95% confidence intervals are shown. All the P-values exceeded 0.05, these were from the moderated t-test results (Smyth, 2004).

According to Robert McMahon, gastropod species of the eulittoral and the littoral fringe pursue different adaptive strategies during low tide emergence: the former keep locomotory and feeding activities and maintain metabolic rates, while the latter withdraw into a shell and onset metabolic inhibition (McMahon, 1988, 1990). The ability of gastropods to quickly slow down metabolic rate after being emerged is critical for successful survival at the littoral fringe (in particular, this limits the expansion of *L. obtusata* to the high shore, Sokolova & Pörtner, 2001). Moreover, littorines enter into short-term metabolic diapause at temperatures above 20-35 °C, which is quite common at exposed stony biotopes of the littoral fringe during the summer season (McMahon *et al*., 1995). Unlike high-shore snails, eulittoral gastropods do not isolate themselves within a shell, but actively locomote and forage during low tide. These are able to use aerial O_2 for breathing. Importantly, these animals are characterized by a very high degree of variability of the $V[O_2]_a : V[O_2]_w$ coefficient (McMahon, 1988). In particular, reported ratios for the *L. obtusata* and *L. saxatilis* (as *rudis*) ranges from 0.59:1 - 7.90:1 and 0.70:1 - 2.60:1, respectively. Such variability in measurements may be a consequence of functional variability of the same species snails: aerial respiratory rates in intertidal gastropods depend in part on a degree of the pallial water retention causing variability in aerial 0_2 uptake rate (Holulihan *et al.*, 1982). Active functioning of the snails during emergence is expectedly accompanied by the loss of the pallial water due to evaporation and consequently by the drop in O_2 uptake rates. This suggests partial anaerobiosis in these snails during the low tide. The functioning of anaerobic metabolism in the low-shore periwinkles under conditions of emergence was confirmed in several populations (Sokolova *et al.*, 2000; Sokolova & Portner, 2001a,b). It was shown as well that the low-shore littorines possess more processive enzymes of anaerobic metabolism, while such enzymes of the high-shore snails are more stable under thermal stress (Sokolova & Portner, 2001a; Panova & Johannesson, 2004). As a result, the low-shore fraction of *L. saxatilis* had a higher abundance of succinate in their foot tissues compared to their high-shore conspecifics in the North Sea populations (Sokolova & Portner, 2001b).

We compared the concentrations of some anaerobic metabolites between inhabitants of the low shore (*L. obtusata*, *L. saxatilis*, *L. compressa*) and the high shore (*L. obtusata*, *L. saxatilis*, *L. arcana*). The

differences in abundances of succinate, lactate and malate were revealed between the high- and low-shore fractions of *L. saxatilis*, as well as between *L. arcana* and *L. compressa* (Fig_8). Unexpectedly, no differences in abundance of amino acids involved in anaerobic metabolism (alanine and asparagine) were found (A6 Fig_4).
